# A chromosome-scale Rhubarb (*Rheum tanguticum*) genome assembly provides insights into the evolution of anthraquinone biosynthesis

**DOI:** 10.1038/s42003-023-05248-5

**Published:** 2023-08-23

**Authors:** Ying Li, Zhenyue Wang, Mingjia Zhu, Zhimin Niu, Minjie Li, Zeyu Zheng, Hongyin Hu, Zhiqiang Lu, Jin Zhang, Dongshi Wan, Qiao Chen, Yongzhi Yang

**Affiliations:** 1 State Key Laboratory of Grassland Agro-Ecosystems, College of Ecology, Lanzhou University, Lanzhou, 730000 China; 2 CAS Key Laboratory of Tropical Forest Ecology, Xishuangbanna Tropical Botanical Garden, Chinese Academy of Sciences, Mengla, Yunnan 666303 China; 3 School of Pharmacy, Lanzhou University, Lanzhou, 730000 China

**Keywords:** Genome duplication, Secondary metabolism, Comparative genomics, Molecular evolution

## Abstract

Rhubarb is the collective name for various perennial plants from the genus *Rheum* L. and the Polygonaceae family. They are one of the most ancient, commonly used, and important herbs in traditional Chinese medicine. Rhubarb is a major source of anthraquinones, but how they are synthesized remains largely unknown. Here, we generate a genome sequence assembly of one important medicinal rhubarb *R. tanguticum* at the chromosome level, with 2.76 Gb assembled into 11 chromosomes. The genome is shaped by two recent whole-genome duplication events and recent bursts of retrotransposons. Metabolic analyses show that the major anthraquinones are mainly synthesized in its roots. Transcriptomic analysis reveals a co-expression module with a high correlation to anthraquinone biosynthesis that includes key chalcone synthase genes. One *CHS*, four *CYP450* and two *BGL* genes involved in secondary metabolism show significantly upregulated expression levels in roots compared with other tissues and clustered in the co-expression module, which implies that they may also act as candidate genes for anthraquinone biosynthesis. This study provides valuable insights into the genetic bases of anthraquinone biosynthesis that will facilitate improved breeding practices and agronomic properties for rhubarb in the future.

## Abstract (web summary)

A chromosome-scale genome assembly for rhubarb combined with transcriptomic data provides insight into the molecular mechanisms regulating anthraquinone biosynthesis in this plant.

## Introduction

**Fig. 1:**
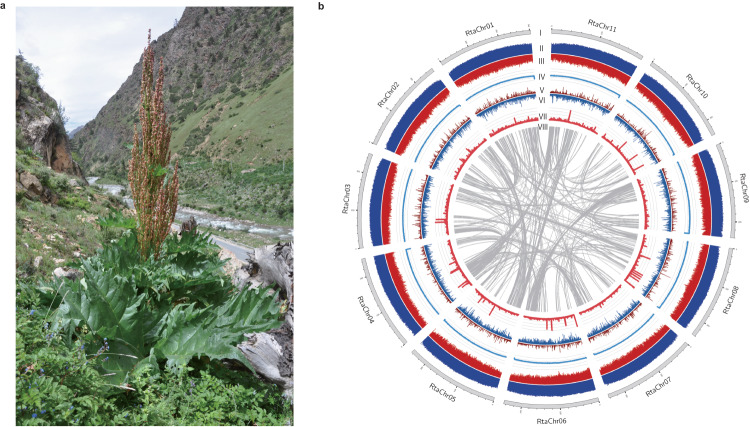
*R. tanguticum* morphology and genome features. **a** Habitat of *R. tanguticum*. **b** Overview of the *R. tanguticum* genome. Different tracks (moving inward) denote (I) chromosomes; (II) density of *Gypsy* elements in 500 kb sliding windows (minimum–maximum, 0–1.0); (III) density of *Copia* elements in 500 kb sliding windows (minimum–maximum, 0–1.0); (IV) GC content in 500 kb sliding windows (minimum–maximum, 0–0.5); (V) repeat density in 500 kb sliding windows (minimum–maximum, 0–1.0); (VI) gene density in 500 kb sliding windows (minimum–maximum, 0–50); (VII) non-coding RNA density in 500 kb sliding windows (minimum–maximum, 0–30); (VIII) identified syntenic blocks.

Rhubarb is an ancient and important herb with thick roots, hollow and erect stems, and small white-green or purple-red flowers clustered along its branches^1^. The name Rhubarb encompasses approximately 60 species of plants in the genus *Rheum* L. from the Polygonaceae family^2^. Rhubarb has mainly been used for medicinal purposes in Asia, though several edible rhubarbs are used in Europe and the Middle East. Of which, the leafstalk of *R. rhabarbarum* is commonly used to make rhubarb pie, which is a traditional dessert in the United States, and is also popular in the Middle East and Canada. In addition, the roots and rhizome of *R. tanguticum* Maxim. and two other species (*R. officinale* Baill. and *R. palmatum* L.) have been officially adopted into both the *Chinese Pharmacopoeia* and *Korean Pharmacopoeia* using the common drug name “Da huang” due to its laxative activity^3^. Among the three medicinal rhubarbs, *R. tanguticum* Maxim. (Fig. 1a) possesses excellent tolerance to alpine environments. In the wild, *R. tanguticum* Maxim. is distributed mainly on the Qinghai–Tibet Plateau and is adjacent to the margins of forest (valleys or shrub meadows), with altitudes ranging from 2300 to 4200 m^4^. It is an important medicinal plant in Northwest China (Gansu, Qinghai, and Tibet) that is beneficial to local economies.

Modern studies of rhubarb have identified its chemical constituents^5,6^, pharmacological activities^7,8^, and functional mechanisms^2,9^ in a more scientific and rigorous way. Extensive photochemistry investigations have led to the isolation and identification of more than 120 compounds from the roots and leaves of rhubarb, which provide chemical evidence for its pharmacological effects^10^. The major biologically-active compounds in rhubarb are a variety of phenolic compounds, including anthraquinones, anthrones, stilbenes, flavonoids, dianthrones, tannins, polyphenols, and chromones^2,11^. While rhubarb is a major source of anthraquinones, the most abundant pharmacological effects in rhubarb are the result of the joint action of several anthraquinones^2^. Anthraquinones are the active components of many traditional medicinal plants that have long been known for their laxative effects^2,12^. For example, in a randomized, double-blind, placebo-controlled clinical trial conducted by Neyrinck et al.^13^, they reported that anthraquinone-rich crude extract supplementation promotes butyrate-producing bacteria and short-chain fatty acid, which is an effective laxative for the treatment of chronic constipation. They also demonstrated that daily oral supplementation of rhubarb extract for 30 days was safe even at higher doses (25 mg per day, calculated as rhein). Another randomized, double-blind, placebo-controlled clinical trial found anthraquinones capsules were used as a safe and effective medicine and showed obvious effects on jaundice with 80 icterohepatitis patients^14^. Moreover, the anthraquinone derivative of rhubarb: emodin^15^, aloe-emodin^16^, rhein^17^, physcion^18^ and chrysophanol^19^ are major biologically-active components that have convincingly demonstrated their abilities to exhibit hepatoprotective, nephroprotective, anti-inflammatory, antioxidant, anticancer, and antimicrobial activities, which lend support to the rationale behind several of its potential medicinal uses. However, more exploration is required into its mechanisms, bioavailability, and safety. In addition, current clinical and commercial use of anthraquinones has also created an urgent demand for its biosynthesis, instead of natural plant extraction.

Anthraquinones are a group of aromatic polyketides that can be synthesized by bacteria, fungi, insects, and plants^20–22^. In plants, anthraquinones are found in a wide range of species, especially in the families Rubiaceae, Polygonaceae, and Rhamnaceae. Biosynthesis of anthraquinone has been mostly studied in Rubiaceae plants, especially in the genera Rubia. These species were known to produce substantial amount of anthraquinone derivatives^12,23^. It has also been reported that the shikimate or chorismateo-succinylbenzoic acid route, which occurs by the addition of succinoylbenzoic acid, is formed from shikimic acid and α-ketoglutaric acid and produces mevalonic acid. This pathway is used to produce anthraquinones with only one hydroxylated ring, such as 1,2-dihydroxylated anthraquinones (Rubia-type anthraquinones), and is commonly used as a natural dye in the textile industry. While the biosynthesis of anthraquinones in rhubarb occurs via a polyketide pathway^24–26^, it produces anthraquinones that are characterized by two hydroxyl groups located on the C-1 and C-8 carbons on its tricyclic aromatic ring (Rhubarb-type anthraquinones). These are known as hydroxyanthraquinones and are characterized as the active components of many traditional medicinal plants. However, how anthraquinones are made via a polyketide pathway remains largely unknown. To date, only a putative Type III polyketide synthase (PKS) gene has been revealed to be responsible for the biosynthesis of an anthraquinone scaffold in a plant (*Senna tora*)^27^. Moreover, although Type III PKS enzymes could actively catalyze seven successive decarboxylative condensations of malonyl-CoA to produce an octaketide chain^26,28^, the linear polyketide chain also undergoes cyclization hydrolysis and decarboxylation to produce the core unit of polyketides, atrochrysone carboxylic acid, which is decarboxylated to atrochrysone with further dehydration and oxidization into emodin anthrone^24,26,28–30^. However, the overall genetic bases for anthraquinone biosynthesis via a polyketide pathway in plants still need further investigation.

Herbgenomics is a new field of study that investigates the genetics and regulatory mechanisms of herbal medicine plants via genomics, which clarifies their mechanisms of action and facilitates molecular breeding from perspective genomes^27,31,32^. Taking a genomics perspective to analyze the metabolic pathways of valuable natural products will yield essential assets for the synthesis and large-scale production of novel chemicals through synthetic biology. Although a rough genome for *Polygonum cuspidatum* (Polygonaceae) has been previously described based on Illumina sequencing^33^, pathways for anthraquinone scaffold biosynthesis and derivatives remain largely elusive due to the low quality of the assembled genome and poor annotation of the relevant genes. Given that *R. tanguticum* is a popular source of rhubarb-type anthraquinones with a wide range of clinical applications and immense potential for drug discovery, in vivo distributions of anthraquinones and their underlying metabolic pathways urgently need to be investigated in this species.

The lack of genomic information for *R. tanguticum* represents a major obstacle in exploring the biological features of rhubarb. To address this problem, we generated a high-quality chromosome-level reference genome for *R. tanguticum* (2*n* = 22) by combining whole-genome shotgun sequencing of Illumina short reads, Oxford Nanopore Technologies (ONT) long reads, and Hi-C data. Together, this represents the first genome of rhubarb. Based on genome evolution analyses, we discovered two recent whole-genome duplication (WGD) events and showed that these WGDs were shared with Tartary buckwheat, another species from the family Polygonaceae. Comparative analysis with other genomes indicated that multiple gene families have expanded in *R. tanguticum*. The WGD-caused expansions in genes that are primarily involved with adaptation to alpine environments, while tandem and proximal duplications caused expansions in genes that may contribute to the notable accumulation of various secondary metabolites in this medicinal plant. Further transcriptome and metabolism analyses revealed a gene co-expression module that is most likely involved in anthraquinone biosynthesis, and we further identified candidate gene sets that may be involved in this pathway. Our study paves the way for the genetic analysis of rhubarb, and gives valuable insights into its genomic characteristics and wide stress tolerance, as well as provides a better understanding of the metabolic pathways of its natural products.

## Results and discussion

### Genome assembly and annotation

A high-quality chromosome-level genome sequence of *Rheum tanguticum* was produced using multiple technologies. In total, 206.84 Gb of Illumina reads (~75× depth), 228.80 Gb of ONT reads (~84× depth), and 296.45 Gb of Hi-C reads (~108× depth) were used to generate this assembly (Supplementary Table 1; depths based on estimated genome size, Supplementary Fig. 1). The primary contig assembly of *R. tanguticum* is larger than the estimated genome size (~3.50 vs. ~2.74 Gb, respectively), which may be due to its high heterozygosity (~1.74%, estimated from *k*-mer frequencies) and high repeat ratio (~85.9%, estimated from *k*-mer frequencies) (Supplementary Fig. 1). After polishing and purging haplotigs, the size of the final *R. tanguticum* assembly (2.76 Gb, N50 = 7.16 Mb; Table 1) was comparable to the estimated genome size. To comprehensively assess the accuracy, continuity and completeness of our *R. tanguticum* genome, four analyses were used to evaluate the assembly quality. In total, the raw Illumina paired-end reads were mapped to the assembled genome with mapping rates of 99.64% (Supplementary Table 2) and the consensus quality value (QV score) was evaluated at 27.8 using Merqury (Supplementary Table 3). Together, these two indices indicate high base accuracies for our *R. tanguticum* genome. Moreover, Benchmarking Universal Single-Copy Orthologs (BUSCO) analysis indicated that 97.3% of the conserved single-copy eukaryotic genes were completely captured in the *R. tanguticum* genome assembly (Supplementary Table 4). Finally, a high long terminal repeat (LTR) Assembly Index (LAI) score of 27.3 were estimated (Supplementary Table 3), which suggested a “golden quality” of rhubarb assembly. Collectively, all four indices highlighted the high quality of our *R. tanguticum* genome assembly.

**Table 1:** Statistics for assembly and annotation of the draft genome of *R. tanguticum*.

	Size
*Assembly*	
Genome size estimate (Gb)	2.74
Heterozygosity (%)	1.74
Genome assembly (Gb)	2.76
Contig N50 (Mb)	7.16
Contig N90 (Mb)	1.91
Longest Contig (Mb)	44.24
Total Contig length (Gb)	2.76
Complete BUSCOs (%)	93.0
*Annotation*	
No. of predicted protein-coding genes	31,898
Average gene length (bp)	3961.62
Average CDS length (bp)	1099.19
Average exon per gene	5.43
Average length of exons (bp)	202.30
Average length of intron (bp)	645.63
Percentage of repeat sequence (%)	87.13
Complete BUSCOs (%)	92.9

The high-depth Hi-C dataset was used to cluster and order the contigs to generate a chromosome-level genome assembly by 3D-DNA pipeline. After the manual correction of the obviously wrong clustering and orientations with Juicebox, we obtained the final chromosome-level assembly (Supplementary Fig. 2). In total, 99.13% of assembled *R. tanguticum* sequences were properly anchored onto 11 chromosomes (Fig. 1b and Supplementary Table 5). The chromatin interactions showed clearly high interaction boundaries between all chromosomes, and linear strong interactions between the close regions within the chromosomes (Supplementary Fig. 2), which both showed a high accuracy of our Hi-C assembly.

A total of 49,000 protein-coding genes were predicted after initial annotation, and then a total of 16,535 pseudogenes and 897 TE-related genes were identified by using PseudogenePipeline and TransposonPSI, respectively. After removing these low-quality genes, a total of 31,898 protein-coding genes were finally obtained (Supplementary Table 6). We have compared the gene characters of the single-copy orthologous between *R. tanguticum* and four other Caryophyllales species (*F. tataricum*, *Simmondsia chinensis, Beta vulgaris* and *Spinacia oleracea*) to validate the quality of our annotation. We found all these five Caryophyllales species showed the similar exon number, CDS length and mRNA length, which suggested the high quality of our gene set (Supplementary Fig. 3). Besides, we also detected the complete BUSCO value of 92.9%, which also showed high completeness of the *R. tanguticum* gene annotation (Supplementary Table 7). About 95.6% of the genes in *R. tanguticum* could be functionally annotated through Blast searches at five functional databases (Supplementary Table 8). In addition, 1876 transcription factors, as well as 10,110 non-coding RNAs (ncRNAs), were identified in *R. tanguticum* (Supplementary Tables 9 and 10).

### Phylogenetic and gene family expansion analyses

**Fig. 2:**
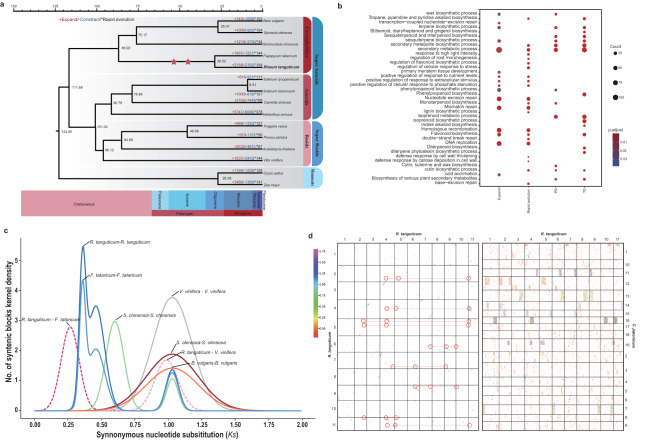
Genome phylogeny and evolutionary dynamics. **a** Phylogenetic tree of *R. tanguticum* and 14 other plant species and dates of WGD events identified in this study (red stars). Gains and losses of gene families in sub-branches are highlighted in red and blue, respectively. **b** Functional enrichment analysis of genes from expanded gene families and genes that were expanded by either TD or PD. The color of each circle represents the statistical significance of enriched GO terms. The size of each circle represents the number of genes within the GO term. “*P* adjust” is the Benjamini–Hochberg false discovery rate (FDR) adjusted *P* value. **c** Distribution of average synonymous substitutions (*Ks*) between syntenic blocks after evolutionary rate correction. **d** Homologous dot plot within *R. tanguticum* genome and between selected *C. japonicum* and *R. tanguticum* chromosomes. The collinear blocks within *R. tanguticum* genome were highlighted in red circles, and the 1:4 syntenic block ratio of the two species was also highlighted by rectangle (one color corresponding to one chromosome of *C. japonicum*).

Gene sequences from 15 species (*R. tanguticum* and four other Caryophyllales, four asterids, four rosids, and two monocots [rice and maize]) were clustered and assigned to 40,758 gene families. Of these, 1110 single-copy gene families were identified and used for phylogenetic analysis (Fig. 2a). *R. tanguticum* was estimated to have diverged from Tartary buckwheat (*Fagopyrum tataricum*, Polygonaceae) ~28.52 million years ago (Mya) (Fig. 2a). Our dating results further indicated that the Polygonaceae species diverged from Amaranthaceae (including beet [*Beta vulgaris*] and spinach [*Spinacia oleracea*]) and Simmondsiaceae (including jojoba [*Simmondsia chinensis*]) ~75.17 Mya, and Caryophyllales diverged from asterids and rosids ~111.68 Mya (Fig. 2a).

Expansion and contraction analysis based on the constructed phylogenetic tree identified 2158, 2155, and 144 gene families that were expanded and contracted and underwent rapid evolution in *R. tanguticum*, respectively (Fig. 2a). According to Gene Ontology (GO) and Kyoto Encyclopedia of Genes and Genomes (KEGG) enrichment analyses, both expanded genes and rapid evolution genes were associated with various secondary metabolite biosynthetic processes, such as “cutin, suberin and wax biosynthesis” (map00073 and GO:0010025), “terpenoid synthesis” (GO:0046246, GO:0051762), “tropane, piperidine and pyridine alkaloid biosynthesis” (map00960) and “flavonoid biosynthesis” (map00940 and GO:0009698) (Fig. 2b). Other gene family expansions were related to DNA damage repair, including “map03430: mismatch repair”, “map03440: homologous recombination”, “map03420: nucleotide excision repair”, and “GO:0006283: transcription-coupled nucleotide-excision repair” (Fig. 2b), which suggest that *R. tanguticum* has enhanced capacities to repair DNA from its colonization of alpine regions^34^. These results imply that the active constituents responsible for the medicinal properties of rhubarb, including massively expanded gene families, are involved in the biosynthesis of various secondary metabolites, as well as mechanisms that respond to stress.

Moreover, we found that 98.3% (5972) of the genes in expanded gene families could be classified into five different categories: 2534 were whole-genome duplicates (WGD duplicates, 41.7%), 705 were tandem duplicates (TD, 8.6%), 408 were proximal duplicates (PD, 6.7%), 852 were transposed duplicates (TRD, 14.0%), and 1651 were dispersed duplicates (27.2%) (Supplementary Fig. 4 and Supplementary Table 11). Although WGD was the primary driver of gene family expansion, these genes were mainly associated with stress response and plant development, which are processes that may relate to its wide distribution and adaptation to high altitudes. However, genes that originate from TD and PD are known to act as important drivers that increase gene product dosage^35^ and accelerate metabolic flux for rate-limiting steps in certain biosynthetic pathways^36^. In agreement with gene family expansion, the expansion of gene families by TD and PD in the *R. tanguticum* genome, showed enrichment of GO categories mainly implicated in secondary metabolite biosynthesis, including for stilbenoid, flavonoid, tropane, and terpenoid biosynthesis pathways (Fig. 2b). In brief, the newly generated tandem and proximal duplications act as the major sources of gene family expansion for medicinally-relevant properties, and each was related to the major constituents of rhubarb, which reflects the biosynthesis of active pharmaceutical ingredients in this medicinal plant^11^. These results suggest that the retention of duplicated genes is an important source of gene family expansion and is responsible for high levels of abiotic stress tolerance that allows for the significant accumulation of secondary metabolites in rhubarb. Ultimately, the genes that originated from TD/PD act as a valuable resource and need further investigation for these biological processes.

### Genome evolution and dynamics

Polyploidizations are thought to be a major driving force in evolution, as it provides additional genetic material that is then more amenable to divergence and adaption^37,38^. To unearth the evolutionary relics from polyploidization in *R. tanguticum*, we first analyzed synonymous substitution rates (*Ks*) of intra-genomic collinear gene pairs within synteny blocks (Supplementary Table 12). Three *Ks* peaks were observed in the two Polygonaceae species, *R. tanguticum* and *F. tataricum* (Tartary buckwheat), which suggests that two rounds of polyploidization event occurred after the γ event (whole-genome triplication, shared by all core eudicots) (Fig. 2a, c). In addition, one *Ks* peak was observed in Amaranthaceae species, spinach, which suggested on recent polyploidization occurred in this species, and all 13 eudicots showed the shared peak of Eudicotcommon hexaploidy (Ech, γ event) (Fig. 2c and Supplementary Fig. 5)^39,40^.

Both intra- or inter-genomic synteny depth analyses were further adopted to reveal the detailed polyploidization histories in the Polygonaceae species (Fig. 2d and Supplementary Figs. 6 and 7). Despite significant gene loss frequently associated with WGD event, fragmental polyploidy relic showed 1:4 chromosomal relationships still present in the majority of chromosomes in both two genomes of Polygonaceae species. For inter-genomic synteny depth analysis, since the beet and spinach genomes underwent complex chromosome rearrangement events^41,42^, we selected *Cercidiphyllum japonicum* and *Vitis vinifera* as our reference genomes because both species have only one polyploidization in their history (the γ event) and few subsequent rearrangements^43^. And we also obtained synteny depth ratios of 4:1 between Polygonaceae species and *C. japonicum*, *V. vinifera*. Both of these results suggested that the two recent round polyploidization events were both WGD (Fig. 2d and Supplementary Figs. 5 and 6). Moreover, to exam these two WGDs were shared by the two Polygonaceae species or not, we performed the following two approaches. First, the collinear genes that showed 4:4 or 4:3 (allow one copy to be lost after WGD) pattern between *R. tanguticum* and *F. tataricum* were extracted to construct the gene trees of each collinear genes group, then the Astral software was used to generate a consensus phylogenetic topology and the quartet-score were further calculated for each internal node (Supplementary Fig. 8). And the results showed that over 84% (144 of 171) gene trees supporting the two WGD events were shared by the two species. Second, the dot plot analyses were also performed between these two species, and the result showed that, for each chromosome region in one species, there are one closest related (lowest *Ks* values) collinear region and three other copied collinear regions in the other species, which also suggested they shared all the WGD events (Supplementary Fig. 9). Our results were different from the published *Fagopyrum* genomes^44,45^ that only detected one recent WGD event only based on the *Ks* distribution result, which also suggested that multiple methods should be applied to reveal the actuary genome evolution^43^.

Genome size also plays a significant role in shaping an organism’s evolution^46–48^ and varies greatly across flowering plants, and is affected by selective pressures imposed by environmental conditions. For example, low levels of atmospheric CO_2_, water availability, and/or the availability of nutrients (N and/or P) favor small genome sizes^48^. We found that *R. tanguticum* has a substantially larger genome than Tartary buckwheat, and is approximately 6x larger in genome size (2.76 vs 0.49 Gb). Since these two species have identical WGD histories, we mainly focused on differences between the two species in abundance of transposable elements (TEs), which usually play a major role in genome size variation between organisms^46,49^. In total, we identified 2.41 Gb of TEs in *R. tanguticum*, which comprises 87.13% of the total genome sequence (Supplementary Table 13 and Supplementary Fig. 8). Long-terminal repeat elements (LTRs) were the most abundant type of TEs and accounted for 94.47% of the total TE sequences in *R. tanguticum* (Supplementary Table 13). *Copia* and *Gypsy* elements were the two most commonly observed families of LTRs and occupied 0.60 Gb and 1.39 Gb in the *R. tanguticum* genome, respectively. Both types of TEs were much more abundant in *R. tanguticum* than Tartary buckwheat (Supplementary Fig. 10), and substantially higher than in other plant genomes^46^. Therefore, substantial accumulation of TEs, especially LTR/*Gypsy* retrotransposons, strongly contributes to a larger difference in genome size between these two species.

TE insertion and removal involve dynamic processes that are influenced by various factors, including natural selection and inherent TE activity^49–51^. We analyzed the accumulation of full-length LTRs and found that they were mainly inserted after the divergence of the two species (Fig. 2a and Supplementary Fig. 10). Both *Copia* and *Gypsy* families burst ~4 Mya in *R. tanguticum* (Supplementary Fig. 10), and the accumulation of TEs in Tartary buckwheat was extremely weak when compared with *R. tanguticum* (Supplementary Fig. 10). Unequal recombination (UR) is another major LTR-RT removal mechanism in plants^50^, the UR between LTRs leads to the removal of intervening sequences and the formation of solo-LTRs. Thus, we further investigated the relative rates of LTR-RT-associated UR as the efficiency of TE removal by measuring the abundance of solo-LTR remnants within the *R. tanguticum* and Tartary buckwheat genomes. These were generated via unequal homologous recombination (HR) events between intact LTRs and can be used as evidence of an inherently efficient DNA removal mechanism. The ratio of solo LTRs to intact LTRs was considerably lower in *R. tanguticum* (i.e., 3.81; 98,465 solo-LTRs: 25,792 intact LTRs) compared to Tartary buckwheat (5.09; 5444: 1069). Thus, the higher frequency of solo-LTRs in Tartary buckwheat may also have contributed to the downsizing of the Tartary buckwheat genome. Altogether, the combination of recent insertion activity and the low efficiency of LTR removal in *R. tanguticum* shaped and maintained its large genome size since the last WGD event.

### Anthraquinone content detection

**Fig. 3:**
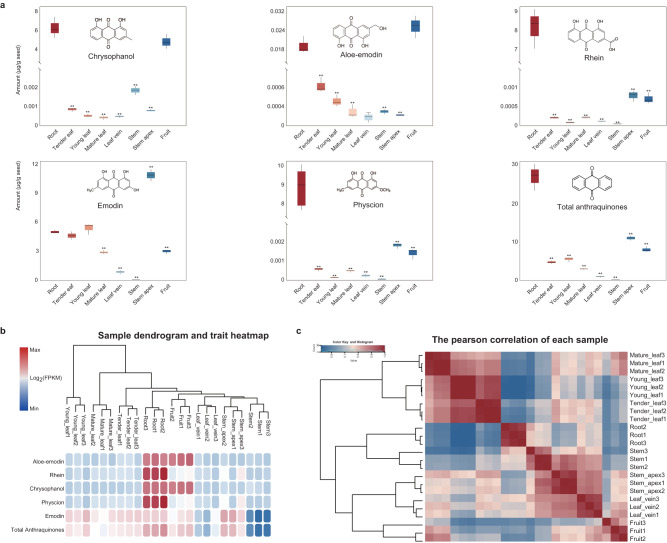
Analysis of anthraquinone contents and transcriptome clustering of eight tissues. **a** Mean concentrations of five anthraquinones within eight different tissues of *R. tanguticum* (*n* = 3 biologically independent samples). **b** Sample dendrogram and trait heatmap indicated the similarity of anthraquinone accumulation patterns among eight tissues. **c** The sample similarity matrix as a reflection of transcriptome-wide gene expression.

One of the main objectives of this study was to dissect potential molecular mechanisms that contribute to anthraquinone biosynthesis and to identify candidate genes in *R. tanguticum*. Here, we measured the in vivo distributions of anthraquinones using targeted metabolomics. We measured the concentrations of five major anthraquinone derivatives (aloe-emodin, rhein, chrysophanol, physcion, and emodin) in eight different tissues, including root, tender leaf, young leaf, mature leaf, leaf vein, stem, stem apex, and fruit using high-performance liquid chromatography technology (Fig. 3a, b). The sample dendrogram and trait heatmap suggest the high repeatability between three independent biological replicates, and our results indicate that these five metabolites were mainly synthesized and accumulated in roots, followed by the stem apex, fruit, and then leaves in different growth stages, which produced similar levels of anthraquinone accumulation. However, leaf veins and stems had the lowest amounts of anthraquinones (Fig. 3a, b). The in vivo distributions of anthraquinones were varied in each tissue, but similar in different leaf developmental stages, and these results are consistent with that the notion that rhubarb root tissue serves as a major source of bioactive metabolite derivatives.

In the root, the total content of anthraquinones (i.e., the total content of the five major anthraquinone derivants detected in this study) (~27 mg g^−1^) was ~2.5× higher than that in the stem apex (~11 mg g^−1^) and ~34× higher than that in the leaf vein (0.8 mg g^−1^). Although the total content of anthraquinones in the stem apex is similar to that of the root, it is mainly due to the high emodin content in the stem apex, since the concentrations of the other four metabolites remained low in the other tissues. The four other anthraquinones showed significantly greater accumulation in the roots, and were 2–3 orders of magnitude higher than the other tissues, especially for rhein and physcion. The concentration of these two anthraquinones was ~8 mg g^−1^ in the roots, but only averaged 0.002 mg g^−1^ in the other seven tissues. The concentration of aloe-emodin was significantly lower than the other four anthroquinones in each type of tissue (average ≤0.06 mg g^−1^). These results revealed that anthraquinones were mainly synthesized in the roots, which is consistent with previous reports^52,53^. Previous studies on rhubarb have only focused on its roots. Here, our study was the first to collect nearly all tissue types from rhubarb, and found abundant accumulation of aloe-emodin in the fruit, and is similar to levels in the roots. We also found that the emodin content in the stem apex was 2× than in the root. Together, these results allow for the specific component extraction of medicinal compounds, which should be used in future drug development.

### Expression pattern analysis of tissue-specific genes

To uncover the key genes involved in the production of anthraquinones, we performed transcriptome analysis to profile the expression patterns of genes across our eight rhubarb tissues (Fig. 3c, *n* = 3 biological replicates). We obtained approximately 7 Gb of clean data for each sample, and over 93% of average reads uniquely aligned to the *R. tanguticum* genome (Supplementary Table 14). In total, 21,206 genes were detected among these tissues with expression levels of fragments per kilobase of transcript per million fragments mapped (FPKM) ≥1 in at least one sample. We found that the samples from the same tissue or from early developmental stages were tightly clustered and exhibited a strong correlation (Fig. 3c).

Based on our anthraquinone contents from the eight tissues, we calculated their differential expression (DEG) by conducting comparative transcriptome analysis between roots and aboveground tissues based on their genome assembly and gene annotation information. Differential expression analysis revealed that there were 11,153 significantly upregulated and 13,871 significantly downregulated genes (false discovery rate [FDR] <0.05) in the roots relative to other tissues (Supplementary Fig. 11). Among these DEGs, there were 821 upregulated and 1354 downregulated genes shared by all of the tissues. To predict the functional roles of the DEGs, we performed GO and KEGG enrichment analyses for each gene that was preferentially expressed in the rhubarb root. GO terms related to root development, such as procambium histogenesis and primary meristem tissue development, were significantly enriched (adjusted *P* < 0.05). In addition, GO terms that included flavonoid biosynthesis were enriched, which are highly associated with the medicinal value of rhubarb (Supplementary Fig. 12).

**Fig. 4:**
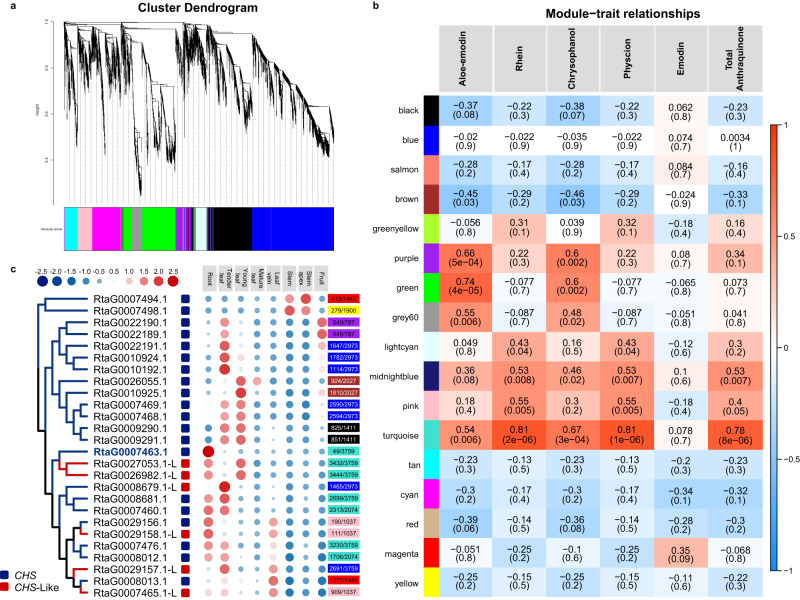
Co-expression analysis to identify groups of genes and the CHS gene family in *R. tanguticum*. **a** Clustering dendrogram shows the co-expression modules recognized by WGCNA. Different colors denote different modules. The longitudinal distance indicates the distance between genes while the horizontal distance is meaningless. **b** Colors on the left represent the 18 modules in the gene co-expression network. For each module, the heatmap shows module eigengene (ME) correlations to traits (content of five anthraquinones and total content of them). Numbers in each cell indicate the correlation coefficients and Student’s asymptotic *P* value (parentheses) for significant ME-trait relationships. Scale bar, right, indicates the range of possible correlations from positive (red, 1) to negative (blue, –1). **c** Phylogenetic tree and expression pattern of CHS genes from *R. tanguticum*. Blue and red rounded rectangles beside the phylogenetic tree indicate classifications of CHS and CHS-like genes, respectively. The expression profiles of the CHS family genes in different tissues are shown in the heatmap. The dot sizes and dot colors represent the different expression levels as illustrated by the legend. Rectangles on the right side and the numbers within them indicate the module color of each gene and its association within its co-expression module, respectively.

These DEGs were further used to identify candidate genes involved with anthraquinone biosynthesis using weighted gene co-expression network analysis (WGCNA). Since anthraquinone biosynthesis mainly occurs in root tissues, co-expression modules were constructed using the expression values of DEGs in the roots. A total of 21,206 DEGs were used in the WGCNA analysis and clustered into 17 modules (Fig. 4a and Supplementary Figs. 13–15). Module-trait relationship analysis revealed that the “turquoise” module contained 3759 genes that were highly correlated with total anthraquinone content (*r* = 0.78, *p* value = 8 × 10^−6^) (Fig. 4b and Supplementary Figs. 13–15). In addition, most genes in the “blue” module were significantly upregulated in root. The “green”, and “purple” modules contained a total of 1530, and 787 genes, respectively, and showed moderate correlations with the content of aloe-emodin and chrysophanol (Fig. 4b and Supplementary Figs. 13–15).

However, most genes in the other modules were significantly upregulated in root, fruit, or tender leaf. Enrichment analyses were also performed for gene sets from these modules, but no related terms were enriched. Because anthraquinone biosynthesis in the plant polyketide pathway is largely unknown, they were not available in the GO or KEGG databases. However, type III PKSs, such as chalcone synthases (*CHS*s), are involved in the biosynthesis of specialized plant metabolites, particularly acetate-pathway-derived flavonoids, stilbenes, and aromatic polyphenols. In the *R. tanguticum* genome, a total of 28 CHS genes were identified, which contained 20 *CHS* and eight *CHS*-L genes. Of these, 26 *CHS* gene with FPKM ≥1 in at least one transcriptome sample (Fig. 4c). Moreover, the *RtaG0007463.1* gene showed the highest expression in the roots and was clustered in the “blue” module where it served as a hub gene (|kME| >0.97) within it. These results indicate that this *CHS* gene had high connectivity in the “turquoise” module and was therefore expected to play an important role in the biosynthesis of anthraquinones (Fig. 4c).

Since TFs play important roles in regulating basic biological processes, we analyzed TF genes that were specifically expressed in the roots to determine whether they function in the regulation of root development in *R. tanguticum*. Indeed, several important transcription factors (TFs) related to the regulation of *CHS* genes and secondary metabolite biosynthesis were clustered in the “turquoise” module. They included seven *bHLH*s genes, which are involved in root hair development and are important regulators of metabolite biosynthesis. A total of 12 *MYB*s were also found clustered in the “turquoise” module, which also are important regulators of metabolite biosynthesis, and two were hub genes. All of these transcription factors interacted with the *CHS* gene, *RtaG0007463.1*. In addition, there are also two *CHS* genes clustered in the “purple” module that are potential candidate genes involved in the biosynthesis of anthraquinones. Together, these results provide a basis for further functional analysis of genes that contribute to the formation of root architecture and the production of bioactive metabolite derivatives in rhubarb roots.

### Identification of candidate gene families for anthraquinone synthesis tailoring

As mentioned above, the linear polyketide chain was generated after successive decarboxylative condensations of eight malonyl-CoA molecules by *CHS* enzymes, which further undergoes a series of modifications (cyclization, hydrolysis, and decarboxylation) to produce the core unit of the anthraquinone scaffold and the final officinal components. However, how anthraquinone precursors are synthesized in plants remains largely unknown, and the subsequent modification of anthraquinone precursors has not been studied yet. Thus, we screened the *R. tanguticum* genome to preliminarily identify candidate gene families for anthraquinone synthesis tailoring.

**Fig. 5:**
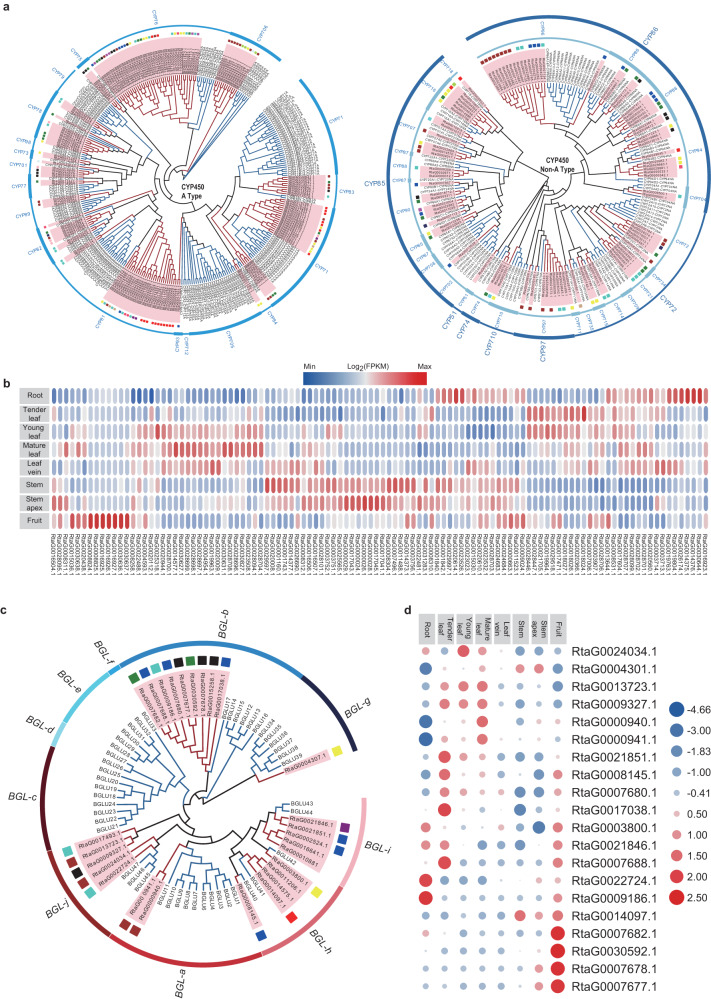
Phylogenetic tree and expression pattern of candidate families for anthraqunine tailoring. **a** Phylogenetic analysis of A-type (left) and non-A-type (right) CYP450 families. The red and blue branches indicate the sequences from *R. tanguticum* and Arabidopsis thaliana, respectively. The red background color of each gene ID also indicates sequences from *R. tanguticum*. The round rectangle beside each gene ID represents the gene’s module color from the WGCNA analysis. The outermost circle indicates the CYP450 gene family. The outermost circle of non-A-type CYP450 phylogenic tree indicates the CYP450 gene family clan. **b** The expression pattern of all A-type CYP450 members. The colored bar indicates the range of expression levels for genes. The colors of the rounded rectangles represent the different expression levels as illustrated by the legend. **c** Phylogenetic tree of BGLs based on the protein sequence alignments from *R. tanguticum* and Arabidopsis. **d** Expression analysis of BGL genes in eight different tissues. The dot sizes and colors represent the different expression levels as illustrated by the legend.

The plant *CYP450* gene family is typically defined as a monooxygenase and plays critical roles in the biosynthesis pathways of secondary metabolites, but they catalyze extremely diverse reactions and have relatively low shared sequence identities^54^. Here, we analyzed *R. tanguticum CYP450* gene families and identified 248 *CYP450* genes using the reported HMM model (PF00067). Together, these genes were divided into two classes: A-type and non-A-type (Fig. 5). The A-type *CYP450s* included only the *CYP71* genes and consisted of 20 families of 153 genes (Fig. 5a), while the non-A-type *CYP450s* contained 12 clans that were composed of 27 families and 95 genes (Fig. 5b). Expression analyses indicated that 172 *CYP450* genes were expressed with average FPKM ≥1. Among these expressed *CYP450* genes, 61 genes exhibited significantly higher expression levels in the root than in the other tissues (FDR <0.01) (Fig. 5c), while there were 83 significantly downregulated *CYPs*. Interestingly, these DEGs included 29 and 28 genes clustered in co-expression modules “turquoise” and “green”, respectively, and both showed expression patterns with high correlations to total anthraquinone content. For example, the four members of the “turquoise” module, *RtaG0030644.1*, *RtaG0014375.1*, *RtaG0014376.1* and *RtaG0026174.1* acted as hub genes for this module, and were highly expressed in the roots (Fig. 5c and Supplementary Table 15). In addition, these hub genes also resided in families that significantly expanded in the *R. tanguticum* genome (Fig. 5a, b). However, other DEGs from the *CYP450* family were considered candidate genes that were not able to be analyzed and need to be studied further in the future. Ultimately, we found that there were an increased number of genes that may encode key enzymes responsible for tailoring anthraquinones synthesis that was coupled with higher transcription in roots that accumulated abundant anthraquinone derivatives. However, these processes complicate their functions in the indigo biosynthesis pathway.

β-Glucosidases (*BGLs*), which belong to the glycoside hydrolase family 1 (GH1), are largely involved in various developmental and stress responses in plants^55–58^. Here, we systematically identified the BGLs in the *R. tanguticum* genome. In total, 27 genes were discovered to encode putative *BGL* genes (Fig. 5c), and phylogenetic analysis of the *BGL*s from *R. tanguticum* and *A. thaliana* showed 10 distinct subgroups, namely, those from *BGL-a* to *BGL-j* (Fig. 5c). However, members from *R. tanguticum* were not detected in subgroups c-f. Gene family analysis also revealed that members from *BGL-b* underwent significant expansion and were thought to be involved in flavonoid utilization^55^. Expression analysis showed that 20 *BGL* members were expressed with an average FPKM ≥1 (Fig. 5d). Among these expressed genes, two members, *RtaG0022724.1* and *RtaG0009186.1*, were expressed significantly higher in the root than the other tissues and clustered in the “turquoise” co-expression module, which may indicate involvement in the biosynthesis of anthraquinones or other secondary metabolites (Fig. 5d). Such genes could be treated as key candidate genes for future functional experiments.

## Concluding remarks

To characterize the evolution of the rhubarb genome and identify candidate genes for anthraquinone biosynthesis, we generated a high-quality chromosome-scale assembly of a key medicinal rhubarb species, *R. tanguticum*, and is the first genome resource of rhubarb. TD and PD-driven gene family expansion may have accelerated the evolution of various secondary metabolite biosynthesis pathways that may also be related to the stress response of this plant. Similar to tandem-arrayed genes in rice, *Arabidopsis* and *Miscanthus lutarioriparius* genomes were enriched in the function of “biotic and abiotic stress”, which retained the duplicated genes as a conservative strategy to adapt to their environments. However, this also makes rhubarb more valuable for medicinal purposes. Our genome evolution analyses unveiled evidence for two WGD events that shared in Polygonaceae lineage. We also found a specific burst of LTR coupled with the genome dynamics associated with a low frequency of LTR removal, which led to genome upsizing in the *R. tanguticum* genome.

One of our main objectives was to dissect potential molecular mechanisms that underly anthraquinone biosynthesis and identify specific genes involved with these processes in *R. tanguticum*. Thus, we combined vast transcriptomic and metabolic data that provide the foundation for rhubarb genomic resources. Based on our multi-omics data, we have identified candidate anthraquinone biosynthesis genes via a polyketide pathway from the *CHS*, *CYP450*, and *BGL* gene families. Together, our resources and results will facilitate the characterization of metabolic pathways, as well as molecular breeding, for this important medicinal plant. Unlike flavonoids, terpenoids, stilbenes or other secondary metabolites whose biosynthesis pathways have been successfully elucidated, the anthraquinone biosynthetic pathways are largely unknown. Together, these candidate genes lay the groundwork for future in vivo experiments that need to further investigate the biosynthesis pathways of anthraquinone.

## Materials and methods

### Genome sample collection and sequencing

Fresh leaf tissue was sampled from a mature wild individual of *R. tanguticum* growing in the Plant Germplasm Repository at Lanzhou University, Gansu Province, China (35°56′30.59″ N, 104°9′16.51″ E, 1747 m) and immediately stored in liquid nitrogen before it was sent to Grandomics (Wuhan, China) for genomic sequencing. High-molecular weight genomic DNA was prepared using the CTAB method and then purified with a QIAGEN^®^ Genomic DNA kit (Cat. No. 13343, QIAGEN). To obtain Illumina short reads, DNA libraries with 500 bp inserts were constructed and sequenced using an Illumina HiSeq 4000 platform. In addition, high-molecular-weight DNA was prepared, and genomic libraries with 20 kb insertions were constructed and sequenced utilizing a PromethION instrument (ONT). The raw reads were filtered using standard criteria (i.e., presence of adapter sequences, low-quality bases, and “mean_qscore <7”). Hi-C (high-throughput chromosome conformation capture) sequencing was performed as follows: sampled DNA was cross-linked with 1% formaldehyde to capture interacting DNA segments, chromatin was digested with the DpnII restriction enzyme, and libraries were constructed and sequenced using the Illumina HiSeq 4000 platform.

### Genome size estimation and assembly

Before estimating genome sizes, short Illumina reads were filtered using fastp (v.0.20.0)^59^ with default parameters. Clean reads were then used to generate *K*-mer (21 bp) frequencies by Jellyfish (v.2.2.10)^60^, and the resulting histogram was exported into GenomeScope (v.1.0.0)^61^. Nextdenovo (v.2.1) (https://github.com/Nextomics/Nextdenovo) was used for correction and de novo assembly of ONT reads with parameters “read_cutoff = 8k, seed_cutoff = 12k, blocksize=8 g, random_round = 100”. The preliminary contigs of *R. tanguticum* were further polished by aligning the Illumina short reads to the contigs using Nextpolish (v.1.1)^62^ in three rounds. Purge Haplotigs^63^ was also applied to remove redundant haplotigs in the *R. tanguticum* genome with the parameter “-a 70”. The quality of the assembly was comprehensively assessed by using four methods: (i) Mapping the Illumina paired-end reads to our final assembly shows high completeness of the genome when high mapping rates are obtained; (ii) BUSCO (v.5.2.1)^64^ was used with the embryophyta_odb10 database and a high percent of complete BUSCOs also indicates high completeness of the genome; (iii) the consensus quality value (QV score) evaluated using Merqury^65^ indicates high base accuracies of the genome with a high QV score; (iv) the LAI evaluated using LTR_retriver^66^ serves as the gold standard for genome benchmarking when LAI >20. Clean Hi-C data were mapped to contig sequences by BWA-MEM (0.7.10-r789)^67^, and valid interaction pairs were extracted. Based on those chromatin interactions, 3D-DNA (v.180922)^68^ was employed to automatically cluster, order, and orient the contigs into pseudo-chromosomes. Juicebox^69^ was used to visualize the chromatin interactions among the assembled pseudo-chromosomes, and then we manually corrected and validated the obvious Hi-C assembly errors to generate the final chromosome assembly.

### Repeat element identification and gene prediction

RepeatMasker (v.4.1.0)^70^ and RepeatProteinMasker (v.4.1.0)^70^ were used to identify repetitive elements in the rhubarb genome based on homology alignments between our assembly sequences and Repbase (v.16.10). We then applied the de novo approach on the rhubarb genome to improve the sensitivity of repeat identification before applying it to our *R. tanguticum* assembly. Briefly, RepeatModeler^71^ and LTR_Finder (v.1.06)^72^ were used to construct a repeat library. Then RepeatMasker^70^ was employed to generate de novo predictions.

A combination of transcriptome-based, homology-based, and de novo-based approaches was used to accurately predict high-quality protein-coding genes. To predict genes ab initio, Augustus (v.3.2.3)^73^, GenScan^74^, and GlimmerHMM (v.3.0.4)^75^ were employed with the *Arabidopsis thaliana* training set. GeMoMa^76^ was used for homology-based prediction, together with protein sequences from *A. thaliana*^77^*, Beta vulgaris*^41^*, Fagopyrum tataricum*^78^*, Prunus persica*^79^*, Vitis vinifera*^80^, and *Spinacia oleracea* (Supplementary Table 16)^42^. For transcriptome-based prediction, de novo transcriptome assemblies were aligned to the genomes to resolve gene structures using PASA. EVidenceModeler (EVM, v.1.1.1)^81^ was then used to generate consensus sets of gene models obtained from the three approaches (transcriptome-based, homology-based, and de novo approaches). To obtain highly reliable gene models, we filtered out single-exon genes supported only by transcriptome-based prediction, as well as those only supported by the ab initio process with fewer than three exons. Although the repeat regions were masked and filtered during gene annotation by de novo approaches, a large number of genes are still unannotated due to the high complexity of this genome. In order to further improve the reliability of our annotated genes, we used TransposonPSI (https://github.com/NBISweden/TransposonPSI) to identify the genes sequence with homology to proteins encoded by diverse families of TEs. In addition, PseudogenePipeline (https://github.com/ShiuLab/PseudogenePipeline) was used to identify the pseudogene. After, the pseudogenes and the TE-related gene with FPKM <1 in the transcriptomic data were excluded from our annotated gene set. For the final protein-coding, functionally annotated genes, they were executed using BLASTP (v.2.7.1+)^82^ (*E* value <1 × 10^−5^) searches against SwissProt and TrEMBL databases. InterProScan (v.5.28)^83^ was then used to annotate protein domains by searching the InterPro databases. GO terms for each gene were obtained from the corresponding InterProScan results. Pathways in which each gene might be involved were assigned using BLAST searches against the KEGG database^84^. Transcription factors in the rhubarb genome were detected using iTAK^85^. ncRNAs were annotated using cmscan from INFERNAL (v1.1.2) (http://eddylab.org/infernal).

### Phylogenetic analysis and expansion/contraction of gene families

To investigate the evolutionary trajectories of *R. tanguticum*, we selected 14 other species for phylogenetic analysis (Supplementary Table 16): *Arabidopsis thaliana*^77^*, Beta vulgaris*^41^*, Camellia sinensis*^86^*, Fragaria vesca*^87^*, Fagopyrum tataricum*^44^*, Helianthus annuus*^88^*, Oryza sativa*^89^*, Prunus persica*^79^*, Simmondsia chinensis*^90^*, Solanum lycopersicum*^91^*, Spinacia oleracea*^42^*, Solanum tuberosum*^92^*, Vitis vinifera*^80^, and *Zea mays*^93^. In order to obtain the orthologous gene set, an all-vs-all BLASTP^82^ search (*E* value cutoff: 1 × 10^−5^) was initially employed to generate similarity information for the genes. We then identified high-quality single-copy genes by applying OrthoMCL (v. 2.0.9-4)^94^ and constructed a concatenation tree and clusters of gene trees using IQ-TREE (v. 2.0.3-h176a8bc_0, with “-m MFP –bb 1000” settings)^95^. We further estimated divergence times between species with MCMCtree (v.4.8) of the PAML package (v.4.8)^96^. Divergence times between *A. thaliana* and *V. vinifera* (115–130 Mya) and *B. vulgaris* and *S. oleracea* (22–30 Mya) were acquired from TimeTree (http://www.timetree.org/) and used as calibration points. Gene family expansions and contractions were further estimated by CAFÉ (v.4.2)^97^ using the gene cluster information and estimated time tree. The parameter λ was estimated along each branch with the random model, and gene families were classified into four types: expanded, contracted, unique, or unchanged.

### Detection of WGD events

In order to reveal the WGD history of *R. tanguticum*, *Ks* distributions, dot plots analyses and phylogenetic analysis of syntenic genes were conducted, refer to the methods from previous procedures published for the *Chloranthus* and *Ceratophyllum*genomes^98,99^. Two Polygonaceae species (*Rheum tanguticum* and *Fagopyrum tataricum*), together with *Spinacia oleracea*, *Vitis vinifera* and *Cercidiphyllum japonicum* were used for WGD analyses. In order to ascertain whether rhubarb and other related species underwent any WGD event, we plotted *Ks* distributions first, reasoning that if recent WGD happend in any species, we would expect *Ks* distributions peak to reflect this as obvious *Ks* peak. Thus, we used WGDI (v.0.5.3)^100^ to identify synteny blocks and collinear genes with “-icl” within each species and between Polygonaceae species. Numbers of synonymous substitutions per synonymous site (*Ks*) between collinear genes were also estimated by “-ks” in WGDI, and a median *Ks* value was selected to represent each syntenic block, with *Ks* peak fitting also performed by WGDI with “-pf”. Second, dot plots of collinear genes and synteny blocks were used to obtain syntenic ratios between the species to confirm the polyploidy level of each species. Moreover, the collinear genes were further extracted and used to construct the gene trees by WGDI with “-a” and “-at” to exam the WGD events were shared between species or not.

### Estimation of TE insertion times and identification of solo-LTR

The dynamic activity of LTR contributes to the vast diversity of genome size and architecture among plants^44,45,47^. For example, LTR expanding over the past million years will lead to the upsizing of a genome, while full-length LTR-RTs with a pair of identical direct repeats (paired-LTRs) favor DNA removal via UR events that lead to the downsizing of the genome. Frequent HR-mediated DNA removal may result in a high abundance of solo-LTR remnants in a genome, which can be used as evidence to prove the existence of an inherently efficient DNA removal mechanism. Therefore, in order to ascertain the effect of LTR dynamics on a genome structure, we estimated the TE insertion times and identified the solo-LTR with the *R. tanguticum* genome. If the *R. tanguticum* genome has undergone a recent burst of LTR and showed inefficient removal of LTR, this would suggest that the dynamic activity of LTR contributes to its large genome size and high repeat ratio, and vice versa.

For estimation of TE insertion times, only LTR sequences identified with a complete 5′-LTR and 3′-LTR were used, since the 5′-LTR is usually identical to the 3′-LTR when a retrotransposon is inserted. The 5′-LTR flanking sequences and 3′-LTR flanking sequences were each aligned using MUSCLE (v.3.8.31)^101^ with default parameters, and evolutionary distances of aligned sequences were calculated using disMat (EMBOSS: v.6.6.0.0, with parameters -nucmethod 2)^102^. Insertion times were calculated using the formula *T* = *K*/2*r*, where *K* represents the divergence between LTRs and r represents the *R. tanguticum* mutation rate of 2.5 × 10^−9^ per base per year.

We used the definition and detection of solo-LTRs and intact LTRs from previous procedures published for the *Welwitschia* genome. Initial LTR-RTs detected by LTR-FINDER were blasted against the “Cores Seq” RefSeqdatabase in *Gypsy* Database v2.0 using blastall (v.2.2.26, with parameters -m 8 -a 4 -F -v 500 -b 250 -e 1e^−5^)^82^. Each blast hit was linked by Solar (version 0.9.6). Alignments were retained when both the coverage and identity were >30%. LTR-RTs with alignments with the “GAG” (Capsid protein), “AP” (Aspartic proteinase), “INT” (Integrase), “RT”, and “RH” (RNaseH) domains were regarded as intact LTR-RTs. Using the LTR sequences (5’LTR or 3’LTR) from intact LTR-RTs, a nucleotide BLAST search was performed against the genome to find potential solo-LTRs. The false solo-LTRs were further filtered by following these criteria: (a) LTRs which overlapped with truncated LTR-RTs; (b) LTRs located within 5 kb of the scaffold edge; (c) LTRs with <0.7 coverage and <0.7 identity cutoff; (d) LTRs identified within 500 bp either side of a gap sequence in the assemblies. To detect truncated LTR-RTs, all LTR-RT sequences reported by LTR-FINDER (v.1.07) were blasted against their genomes, and alignments with >80% coverage and >60% identity were considered to correspond to the presence of truncated LTR-RTs.

### Transcriptome sequencing and analysis

To assist gene predictions and dissect the molecular basis that underlies anthraquinone biosynthesis in *R. tanguticum*, we performed transcriptome sequencing for eight different tissues, including root, tender leaves, young leaves, mature leaves, leaf veins, stems, stem apexes, and fruits. Three biological replicates were used for each sample. Total RNA extraction, library construction, and sequencing were performed by BGI-Shenzhen Company (Wuhan, China) using an MGI2000 platform with 2 × 150 bp paired-end runs. After filtering low-quality reads by fastp, clean reads were mapped to the *R. tanguticum* genome assembly using HISAT2 (v.2.2.1)^103^. StringTie (v.2.1.2)^104^ was used to predict new transcripts, which were combined with gene annotations to obtain a final transcriptome set. DEseq2 (v.1.22.2)^105^ was used to identify DEGs, defined as those with |log2(fold change)| >1 and FDR significance score (*P*_adj_) <0.05. DEGs were subjected to KEGG and GO enrichment analysis using clusterProfiler^106^. Gene co-expression networks were constructed using the WGCNA^107^ package in the R software. The core DEGs were further divided into three modules using WGCNA, and correlations of each module with anthraquinone contents were calculated. Module-trait associations were estimated using the correlation between the module eigengene and root/control treatments. A signed network was constructed in WGCNA with specific parameter settings of power = 9, networkType = “signed”, TOMType = “unsigned”, and minModuleSize = 200.

### Determination of metabolite concentrations

We collected fresh tissues from the roots, tender leaves, young leaves, mature leaves, leaf veins, stems, stem apexes, and fruits, and determined the concentrations of aloe-emodin, rhein, chrysophanol, physcion, and emodin in *R. tanguticum*. Briefly, these tissues were immediately frozen in liquid nitrogen, and metabolites were extracted from about 0.1 g of material with 1.5 ml of methanol-2 mM ammonium formate solution (9:1) followed by vortex oscillation for 1 min and grinding for 3 min. Next, ultrasonic oscillation was performed for 40 min, followed by vortexing for 30 s and then a 1-h incubation at 4 °C. The solution was then centrifuged at 4 °C for 15 min at 12,000 rpm, and the aqueous layer was filtered through a 0.22 μm filter membrane. Three replicate samples were prepared for each tissue type. The concentrations of these five compounds were determined using a high-performance liquid chromatography system. Three replicates of each tissue were performed^27^.

### Analysis of *CHS, CYP450*, and *BGL* gene families

The members from the *CHS*, *CYP450*, and *BGL* gene families are probably involved in the production of anthraquinones^24–26^. Thus, we identified all the members of these gene families at the genome-wide level in *R. tanguticum*. For the identification and classification of *CHS* genes, hmmsearch was used to identify them in the *R. tanguticum* genome using PF02797 and PF00195 from the Pfam database. *CHS* genes from *Senna tora* were also used as query sequences against the *R. tanguticum* protein database via BLASTP searches (*e* value of 1e-5, >40% identity value, and >40% coverage). The candidate *CHS* genes were further classified by integrity, and the *CHS* genes with one or two fragmentary domains were identified as *CHS*-like genes. For the identification and classification of *CYP450* genes, hmmsearch^108^ was used by PF00067 from the Pfam database. We also downloaded the *Arabidopsis CYP450* protein sequences from the website (http://www.p450.kvl.dk/). These proteins were then used as query sequences against the *R. tanguticum* protein database using BLASTP with same parameters as above. The classification of the CYP450 genes was performed by alignment with the *CYP450* database using standard sequence similarity cut-offs, with definite standards of 97%, 55%, and 40% for allelic, subfamily, and family variants, respectively. According to the standardized *CYP450* nomenclature, *CYP450s* were divided into A-type and non-A-type *CYP450s*, and phylogenetic analysis of *CYP450* genes was performed for A-type and non-A-type *CYP450s*. The protein sequences of *BGL* members were downloaded from TAIR (http://www.arabidopsis.org/tools/bulk/sequences/index.jsp). To identify BGL family members, PF00232 from the Pfam database was used to query all putative protein sequences of *R. tanguticum* using hmmsearch. Genes from each gene family were aligned using MAFFT^109^, and the resulting alignment was then delivered to IQ-TREE to construct a phylogenetic tree.

### Statistics and reproducibility

The functional enrichment analysis was performed using the ClusterProfile. The statistical significance of GO terms was evaluated using Fisher’s exact test in combination with FDR correction for multiple testing (*P* < 0.05). All experiments were carried out at least three times, independently, with similar results. All values are presented as means ± SD. Statistical significance was based on *t*-tests.

### Reporting summary

Further information on research design is available in the Nature Portfolio Reporting Summary linked to this article.

### Supplementary information

Supplementary Figure and Tables

Reporting summary

## Data Availability

The genome assembly file and genome annotation files (contig level and chromosome level) are available at Figshare (10.6084/m9.figshare.19663062). All genomic data (short-reads sequencing data, long-reads sequencing data, and Hi-C sequencing data) have been deposited at NCBI under the BioProject accession number PRJNA746014. All transcriptome data have been deposited at NGDC under the BioProject accession number PRJCA009275. The source data behind the graphs in Figs. 2b, c and 3a are available at Figshare (10.6084/m9.figshare.19663062) as Supplementary Data 1–3, respectively. All other data are available from the corresponding authors upon reasonable request.

## References

[1] Lee M, Hutcheon J, Dukan E, Milne I (2017). Rhubarb (*Rheum* Species): the role of Edinburgh in its cultivation and development. J. R. Coll. Physicians Edinb..

[2] Cao Y-J (2017). Advances in bio-active constituents, pharmacology and clinical applications of rhubarb. Chin. Med..

[3] VanMen C (2012). Chemical-based species classification of rhubarb using simultaneous determination of five bioactive substances by HPLC and LDA analysis. Phytochem. Anal..

[4] Tan, L., Geng, D., Hu, F. & Dong, Q. Rapid identification and quantification of natural antioxidants in the seeds of Rhubarb from different habitats in China using accelerated solvent extraction and HPLC-DAD-ESI–MS ^*n*^-DPPH Assay. *J. Chromatogr. Sci.* **54**, 48–57 (2016). 10.1093/chromsci/bmv105 26206792

[5] Jin W (2006). Development of high-performance liquid chromatographic fingerprint for the quality control of Rheum tanguticum Maxim. ex Balf. J. Chromatogr. A.

[6] Luo D (2021). Integrating the rapid constituent profiling strategy and multivariate statistical analysis for herb ingredients research, with Chinese official rhubarb and Tibetan rhubarb as an example. Arab. J. Chem..

[7] Chen D, Wang L (2009). Mechanisms of therapeutic effects of rhubarb on gut origin sepsis. Chin. J. Traumatol..

[8] Chen D, Ma L, Liu S (2009). Effects of rhubarb on intestinal flora and bacterial translocation in rats with sepsis. Zhongguo Wei Zhong Bing. Ji Jiu Yi Xue.

[9] Chen J-Q (2020). An integrated metabolomics strategy to reveal dose-effect relationship and therapeutic mechanisms of different efficacy of rhubarb in constipation rats. J. Pharm. Biomed. Anal..

[10] Wang, Y. U. et al. Research progress on chemical composition and pharmacological effects of Rhei Radix et Rhizoma and predictive analysis on quality markers. *Chin. Tradit. Herb. Drugs* **50**, 4821–4837 (2019).

[11] Xiang H, Zuo J, Guo F, Dong D (2020). What we already know about rhubarb: a comprehensive review. Chin. Med.

[12] Diaz-Muñoz, G., Miranda, I. L., Sartori, S. K., de Rezende, D. C. & Diaz, M. A. N. Chapter 11 – Anthraquinones: an overview. in *Studies in Natural Products Chemistry* (ed. Atta-ur-Rahman) 58, 313–338 (Elsevier, 2018).

[13] Neyrinck, A. M. et al. Constipation mitigation by Rhubarb extract in middle-aged adults is linked to gut microbiome modulation: a double-blind randomized placebo-controlled trial. *Int. J. Mol. Sci.* **23**, 14685 (2022). 10.3390/ijms232314685 PMC9738964 36499011

[14] Guo D (2016). Clinical observation on the total anthraquinones of rhubarb. Clin. J. Chin. Med..

[15] Dong X (2016). Emodin: a review of its pharmacology, toxicity and pharmacokinetics. Phytother. Res..

[16] Dong X (2020). Aloe-emodin: a review of its pharmacology, toxicity, and pharmacokinetics. Phytother. Res..

[17] Zhou Y-X (2015). Rhein: a review of pharmacological activities. Evid. Based Complement. Altern. Med..

[18] XunLi (2019). Physcion and physcion 8-O-β-glucopyranoside: a review of their pharmacology, toxicities and pharmacokinetics. Chem. Biol. Interact..

[19] Su S (2020). The pharmacological properties of chrysophanol, the recent advances. Biomed. Pharmacother..

[20] Shamim G, Ranjan SK, Pandey DM, Ramani R (2014). Biochemistry and biosynthesis of insect pigments. Eur. J. Entomol..

[21] Chiang Y-M (2010). Characterization of the *Aspergillus nidulans* monodictyphenone gene cluster. Appl. Environ. Microbiol..

[22] Zhou H, Li Y, Tang Y (2010). Cyclization of aromatic polyketides from bacteria and fungi. Nat. Prod. Rep..

[23] Malik EM, Müller CE (2016). Anthraquinones as pharmacological tools and drugs. Med. Res. Rev..

[24] Abdel-Rahman IAM (2013). In vitro formation of the anthranoid scaffold by cell-free extracts from yeast-extract-treated *Cassia bicapsularis* cell cultures. Phytochemistry.

[25] Foyer CH, Noctor G (2011). Ascorbate and glutathione: the heart of the Redox Hub1. Plant Physiol..

[26] Mizuuchi Y (2009). Novel type III polyketide synthases from Aloe arborescens. FEBS J..

[27] Kang S-H (2020). Genome-enabled discovery of anthraquinone biosynthesis in Senna tora. Nat. Commun..

[28] Karppinen K, Hokkanen J, Mattila S, Neubauer P, Hohtola A (2008). Octaketide-producing type III polyketide synthase from *Hypericum perforatum* is expressed in dark glands accumulating hypericins. FEBS J..

[29] Abe I, Oguro S, Utsumi Y, Sano Y, Noguchi H (2005). Engineered biosynthesis of plant polyketides: chain length control in an octaketide-producing plant type III polyketide synthase. J. Am. Chem. Soc..

[30] Pillai PP, Nair AR (2014). Hypericin biosynthesis in Hypericum hookerianum Wight and Arn: investigation on biochemical pathways using metabolite inhibitors and suppression subtractive hybridization. C. R. Biol..

[31] Wuyun T (2018). The hardy rubber tree genome provides insights into the evolution of polyisoprene biosynthesis. Mol. Plant.

[32] Kang M (2020). A chromosome-scale genome assembly of Isatis indigotica, an important medicinal plant used in traditional Chinese medicine: an Isatis genome. Hortic. Res.

[33] Zhang Y (2019). Assembly and annotation of a draft genome of the medicinal plant *Polygonum cuspidatum*. Front. Plant Sci..

[34] Hu Y (2022). The potential roles of unique leaf structure for the adaptation of *Rheum tanguticum* Maxim. ex Balf. in Qinghai–Tibetan Plateau. Plants.

[35] Conant GC, Wolfe KH (2008). Turning a hobby into a job: how duplicated genes find new functions. Nat. Rev. Genet..

[36] Bekaert M, Edger PP, Pires JC, Conant GC (2011). Two-phase resolution of polyploidy in the Arabidopsis metabolic network gives rise to relative and absolute dosage constraints. Plant Cell.

[37] Otto SP (2007). The evolutionary consequences of polyploidy. Cell.

[38] Soltis PS, Marchant DB, Van de Peer Y, Soltis DE (2015). Polyploidy and genome evolution in plants. Curr. Opin. Genet. Dev..

[39] Jiao Y (2012). A genome triplication associated with early diversification of the core eudicots. Genome Biol..

[40] Vekemans D (2012). Gamma paleohexaploidy in the stem lineage of core eudicots: significance for MADS-box gene and species diversification. Mol. Biol. Evol..

[41] Dohm JC (2014). The genome of the recently domesticated crop plant sugar beet (*Beta vulgaris*). Nature.

[42] Xu C (2017). Draft genome of spinach and transcriptome diversity of 120 *Spinacia accessions*. Nat. Commun..

[43] Wang Z (2022). A high-quality Buxus austro-yunnanensis (Buxales) genome provides new insights into karyotype evolution in early eudicots. BMC Biol..

[44] Zhang L (2017). The tartary buckwheat genome provides insights into rutin biosynthesis and abiotic stress tolerance. Mol. Plant.

[45] He M (2022). Comparison of buckwheat genomes reveals the genetic basis of metabolomic divergence and ecotype differentiation. N. Phytol..

[46] Wang D (2021). Which factors contribute most to genome size variation within angiosperms?. Ecol. Evol..

[47] Blommaert J (2020). Genome size evolution: towards new model systems for old questions. Proc. R. Soc. B..

[48] Faizullah L (2021). Exploring environmental selection on genome size in angiosperms. Trends Plant Sci..

[49] Zhang S-J, Liu L, Yang R, Wang X (2020). Genome size evolution mediated by gypsy retrotransposons in brassicaceae. Genom. Proteom. Bioinforma..

[50] Niu S (2022). The Chinese pine genome and methylome unveil key features of conifer evolution. Cell.

[51] Wan T (2021). The Welwitschia genome reveals a unique biology underpinning extreme longevity in deserts. Nat. Commun..

[52] Liu, J. et al. Main components analysis in different parts of Rheum palmatum. *Chin. Tradit. Herb. Drugs* **48**, 567–572 (2017).

[53] Chen, Y.-Y. Research progress and utilization strategy on resource chemistry of Rhei Radix et Rhizoma. *Chin. Tradit. Herb. Drugs* **49**, 5170–5178 (2018).

[54] Yu J (2017). Evolutionary history and functional divergence of the cytochrome P450 gene superfamily between *Arabidopsis thaliana* and Brassica species uncover effects of whole genome and tandem duplications. BMC Genom..

[55] Xu Z (2004). Functional genomic analysis of *Arabidopsis thaliana* glycoside hydrolase family 1. Plant Mol. Biol..

[56] Chandrasekar B (2014). Broad-range glycosidase activity profiling. Mol. Cell. Proteom..

[57] Henrissat B (1991). A classification of glycosyl hydrolases based on amino acid sequence similarities. Biochem. J..

[58] Opassiri R (2006). Analysis of rice glycosyl hydrolase family 1 and expression of Os4bglu12 β-glucosidase. BMC Plant Biol..

[59] Chen S, Zhou Y, Chen Y, Gu J (2018). fastp: an ultra-fast all-in-one FASTQ preprocessor. Bioinformatics.

[60] Marçais G, Kingsford C (2011). A fast, lock-free approach for efficient parallel counting of occurrences of k-mers. Bioinformatics.

[61] Vurture GW (2017). GenomeScope: fast reference-free genome profiling from short reads. Bioinformatics.

[62] Hu J, Fan J, Sun Z, Liu S (2020). NextPolish: a fast and efficient genome polishing tool for long-read assembly. Bioinformatics.

[63] Roach MJ, Schmidt SA, Borneman AR (2018). Purge Haplotigs: allelic contig reassignment for third-gen diploid genome assemblies. BMC Bioinforma..

[64] Simão FA, Waterhouse RM, Ioannidis P, Kriventseva EV, Zdobnov EM (2015). BUSCO: assessing genome assembly and annotation completeness with single-copy orthologs. Bioinformatics.

[65] Rhie A, Walenz BP, Koren S, Phillippy AM (2020). Merqury: reference-free quality, completeness, and phasing assessment for genome assemblies. Genome Biol..

[66] Ou S, Jiang N (2018). LTR_retriever: a highly accurate and sensitive program for identification of long terminal repeat retrotransposons. Plant Physiol..

[67] Li H, Durbin R (2009). Fast and accurate short read alignment with Burrows–Wheeler transform. Bioinformatics.

[68] Dudchenko O (2017). De novo assembly of the *Aedes aegypti* genome using Hi-C yields chromosome-length scaffolds. Science.

[69] Durand NC (2016). Juicer provides a one-click system for analyzing loop-resolution Hi-C experiments. Cell Syst..

[70] Tarailo-Graovac M, Chen N (2009). Using RepeatMasker to identify repetitive elements in genomic sequences. Curr. Protoc. Bioinforma..

[71] Bao W, Kojima KK, Kohany O (2015). Repbase Update, a database of repetitive elements in eukaryotic genomes. Mob. DNA.

[72] Xu Z, Wang H (2007). LTR_FINDER: an efficient tool for the prediction of full-length LTR retrotransposons. Nucleic Acids Res..

[73] Stanke M, Morgenstern B (2005). AUGUSTUS: a web server for gene prediction in eukaryotes that allows user-defined constraints. Nucleic Acids Res..

[74] Burge C, Karlin S (1997). Prediction of complete gene structures in human genomic DNA. J. Mol. Biol..

[75] Majoros WH, Pertea M, Salzberg SL (2004). TigrScan and GlimmerHMM: two open source ab initio eukaryotic gene-finders. Bioinformatics.

[76] Keilwagen J, Hartung F, Grau J (2019). GeMoMa: homology-based gene prediction utilizing intron position conservation and RNA-seq data. Methods Mol. Biol..

[77] Zapata, L. et al. Chromosome-level assembly of *Arabidopsis thaliana* L *er* reveals the extent of translocation and inversion polymorphisms. *Proc. Natl. Acad. Sci. USA*. **113**, E4052–E4060 (2016). 10.1073/pnas.1607532113 PMC4948326 27354520

[78] Matsui K, Yasui Y (2020). Buckwheat heteromorphic self-incompatibility: genetics, genomics and application to breeding. Breed. Sci..

[79] Verde I (2017). The Peach v2.0 release: high-resolution linkage mapping and deep resequencing improve chromosome-scale assembly and contiguity. BMC Genom..

[80] The French–Italian Public Consortium for Grapevine Genome Characterization. (2007). The grapevine genome sequence suggests ancestral hexaploidization in major angiosperm phyla. Nature.

[81] Haas BJ (2008). Automated eukaryotic gene structure annotation using EVidenceModeler and the Program to Assemble Spliced Alignments. Genome Biol..

[82] Altschul SF (1997). Gapped BLAST and PSI-BLAST: a new generation of protein database search programs. Nucleic Acids Res..

[83] Quevillon E (2005). InterProScan: protein domains identifier. Nucleic Acids Res..

[84] Ogata H (1999). KEGG: Kyoto Encyclopedia of Genes and Genomes. Nucleic Acids Res..

[85] Zheng Y (2016). iTAK: a program for genome-wide prediction and classification of plant transcription factors, transcriptional regulators, and protein kinases. Mol. Plant.

[86] Xia E-H (2017). The tea tree genome provides insights into tea flavor and independent evolution of caffeine biosynthesis. Mol. Plant.

[87] Buti M (2017). The genome sequence and transcriptome of *Potentilla micrantha* and their comparison to *Fragaria vesca* (the woodland strawberry). Gigascience.

[88] Badouin H (2017). The sunflower genome provides insights into oil metabolism, flowering and Asterid evolution. Nature.

[89] Goff SA (2002). A draft sequence of the rice genome (*Oryza sativa* L. ssp. japonica). Science.

[90] Sturtevant D (2020). The genome of jojoba (*Simmondsia chinensis*): a taxonomically isolated species that directs wax ester accumulation in its seeds. Sci. Adv..

[91] The Tomato Genome Consortium. (2012). The tomato genome sequence provides insights into fleshy fruit evolution. Nature.

[92] Barchi L (2019). A chromosome-anchored eggplant genome sequence reveals key events in Solanaceae evolution. Sci. Rep..

[93] Jiao Y (2017). Improved maize reference genome with single-molecule technologies. Nature.

[94] Li L, Stoeckert CJ, Roos DS (2003). OrthoMCL: identification of ortholog groups for eukaryotic genomes. Genome Res..

[95] Nguyen L-T, Schmidt HA, von Haeseler A, Minh BQ (2015). IQ-TREE: a fast and effective stochastic algorithm for estimating maximum-likelihood phylogenies. Mol. Biol. Evol..

[96] Yang Z (2007). PAML 4: phylogenetic analysis by maximum likelihood. Mol. Biol. Evol..

[97] De Bie T, Cristianini N, Demuth JP, Hahn MW (2006). CAFE: a computational tool for the study of gene family evolution. Bioinformatics.

[98] Ma J (2021). The *Chloranthus sessilifolius* genome provides insight into early diversification of angiosperms. Nat. Commun..

[99] Yang Y (2020). Prickly waterlily and rigid hornwort genomes shed light on early angiosperm evolution. Nat. Plants.

[100] Sun, P. et al. WGDI: a user-friendly toolkit for evolutionary analyses of whole-genome duplications and ancestral karyotypes. *Mol. Plant* **15**, 208–222 (2021). 10.1016/j.molp.2022.10.018 36307977

[101] Edgar RC (2004). MUSCLE: multiple sequence alignment with high accuracy and high throughput. Nucleic Acids Res..

[102] Rice P, Longden I, Bleasby A (2000). EMBOSS: the European Molecular Biology Open Software Suite. Trends Genet.

[103] Kim D, Langmead B, Salzberg SL (2015). HISAT: a fast spliced aligner with low memory requirements. Nat. Methods.

[104] Pertea M (2015). StringTie enables improved reconstruction of a transcriptome from RNA-seq reads. Nat. Biotechnol..

[105] Love MI, Huber W, Anders S (2014). Moderated estimation of fold change and dispersion for RNA-seq data with DESeq2. Genome Biol..

[106] clusterProfiler: an R Package for Comparing Biological Themes Among Gene Clusters. https://www.liebertpub.com/doi/epdf/10.1089/omi.2011.0118 or 10.1089/omi.2011.0118. 10.1089/omi.2011.0118 PMC3339379 22455463

[107] Langfelder P, Horvath S (2008). WGCNA: an R package for weighted correlation network analysis. BMC Bioinforma..

[108] Johnson LS, Eddy SR, Portugaly E (2010). Hidden Markov model speed heuristic and iterative HMM search procedure. BMC Bioinforma..

[109] Katoh K, Misawa K, Kuma K, Miyata T (2002). MAFFT: a novel method for rapid multiple sequence alignment based on fast Fourier transform. Nucleic Acids Res..

