# Supplementary material for: A chromosome-scale Rhubarb (Rheum tanguticum) genome assembly provides insights into the evolution of anthraquinone biosynthesis
Source: Commun Biol. 2023 Aug 23;6:867. doi: 10.1038/s42003-023-05248-5 (PMC10447539; doi:10.1038/s42003-023-05248-5)
Supplement: Supplementary file 1 — Supplementary Figure and Tables [file 42003_2023_5248_MOESM1_ESM.pdf]

Supplementary Figures

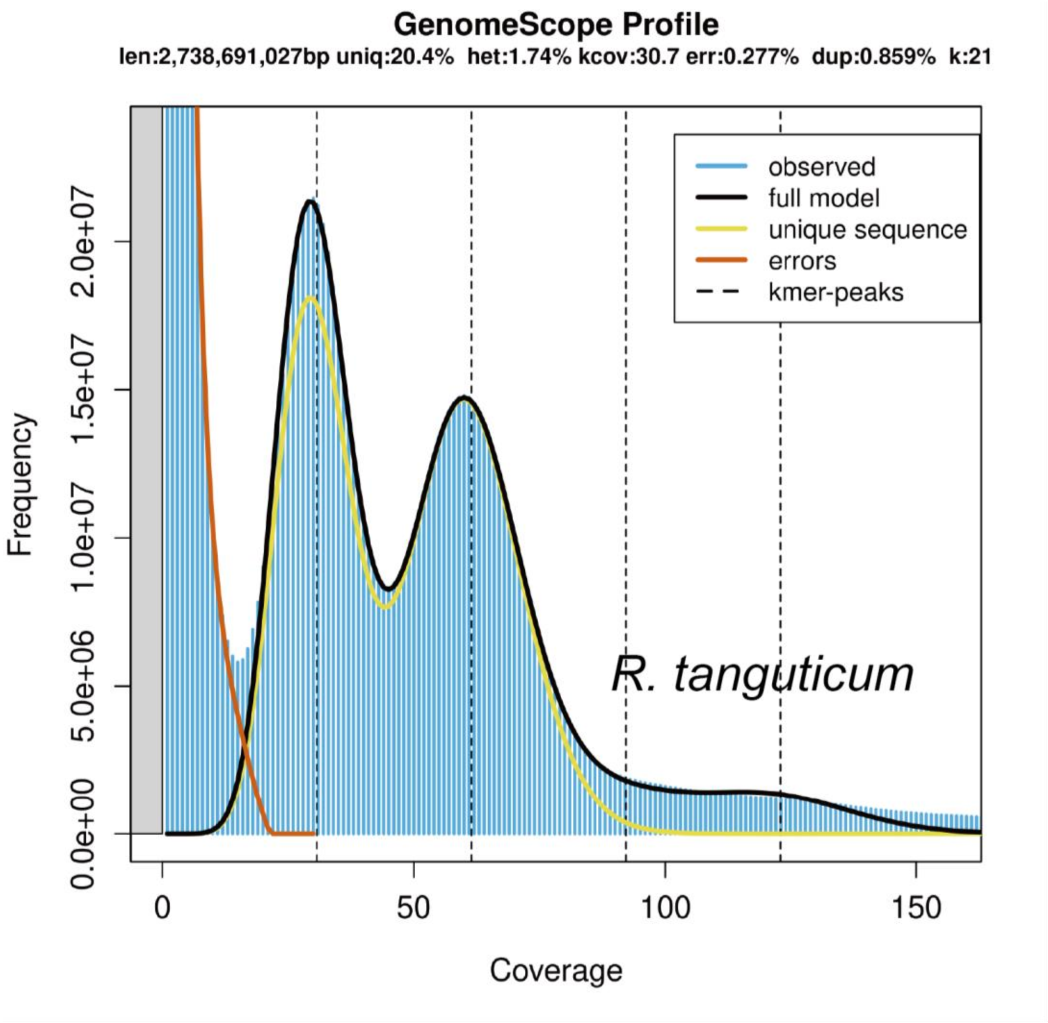

Supplementary Figure 1. Genome survey of *R. tanguticum* based on GenomeScope with the *K*-mer size of 21. A *K*-mer refers to an artificial sequence division of *K* nucleotides from reads. Genomic characteristics (genome size, repeat structure, and heterozygous rate) could be estimated based on *K*-mer frequencies. Blue solid line for observed *K*-mer frequency distribution, red dash line for fitted model of *K*-mer frequency distribution.

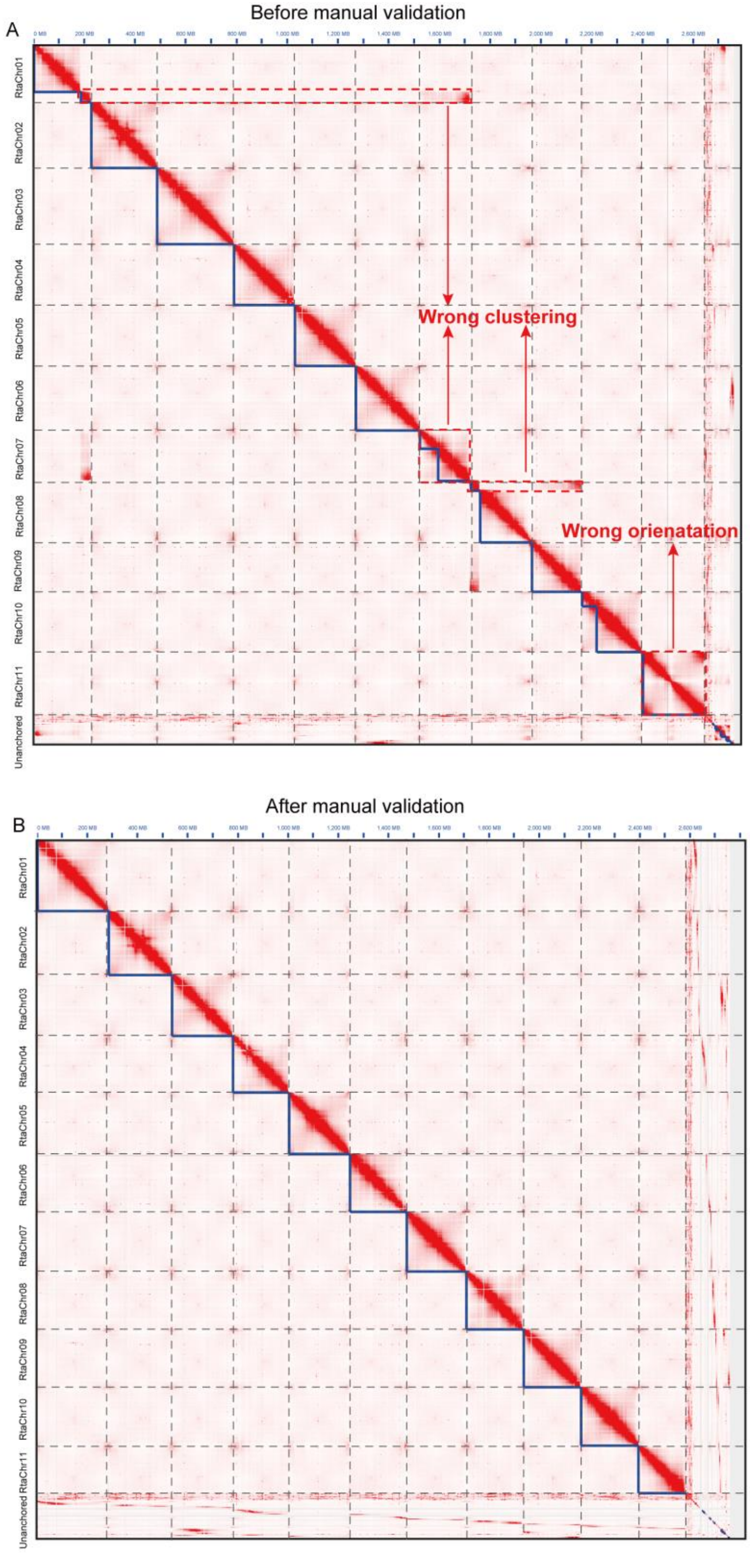

Supplementary Figure 2. Genome-wide analysis of chromatin interactions in the chromosome-level genome assemblies of *R. tanguticum* based on Hi-C data. (A) Raw Hi-C scaffolding results generated by 3D-DNA and the example of obvious clustering/orientation errors. (B) The final version of *R. tanguticum* chromosomes scaffolding results after manual validation.

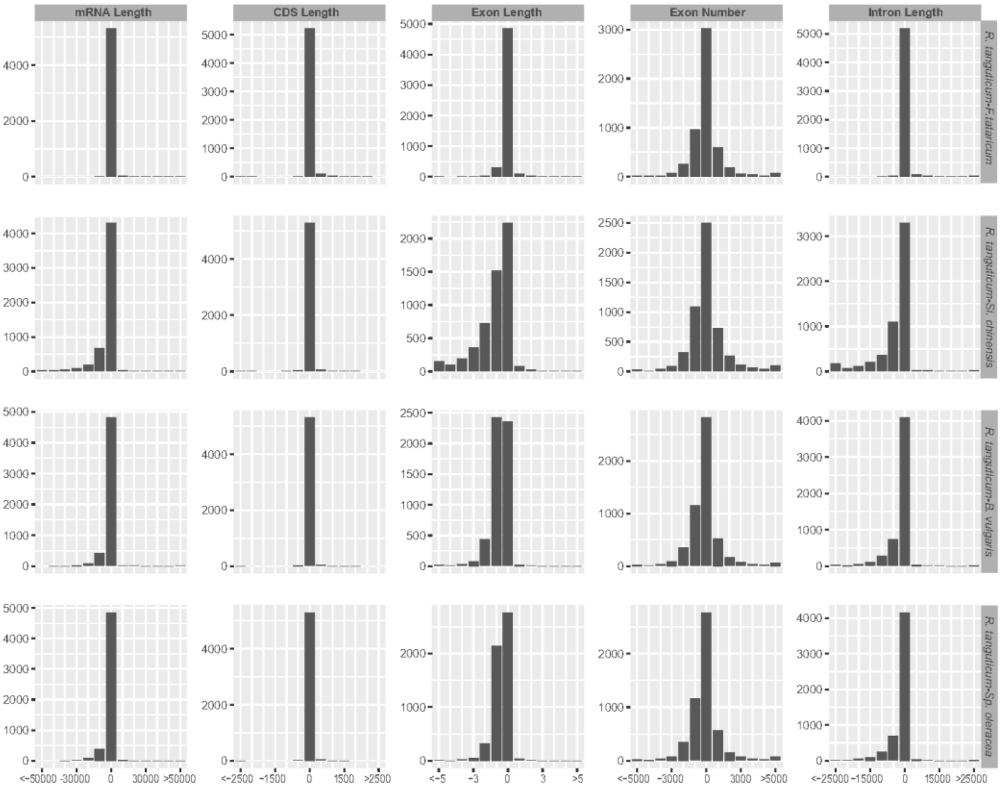

Supplementary Figure 3. The length difference and number of exons difference between the orthologous gene pair of *R. tanguticum* and four other Caryophyllales (*Fagopyrum tataricum, Simmondsia chinensis, Beta vulgaris* and *Spinacia oleracea*).

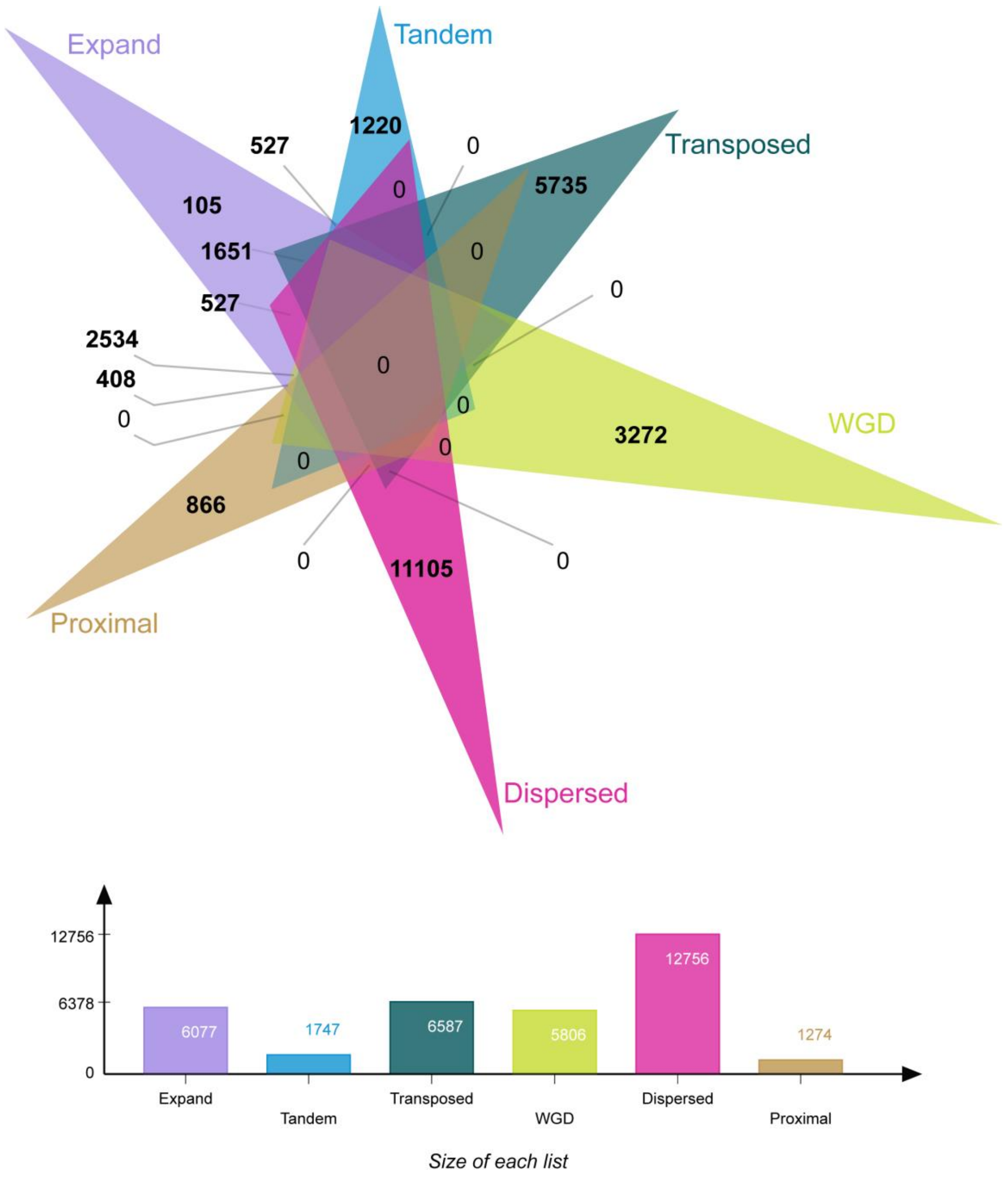

Supplementary Figure 4. Classification of gene duplicates origin in *R. tanguticum* genome. (a) Histogram shows the origins of gene duplicates were classified into five types: whole genome duplication, tandem duplication (WGD), proximal duplication (PD), dispersed duplication (DSD), and tandem duplication (TD) and transposed duplication (TRD); **(b)** Venn diagram shows the possible logical relations between members of expanded gene family and modes of duplication (WGD, TD, PD, TRD, and DSD).

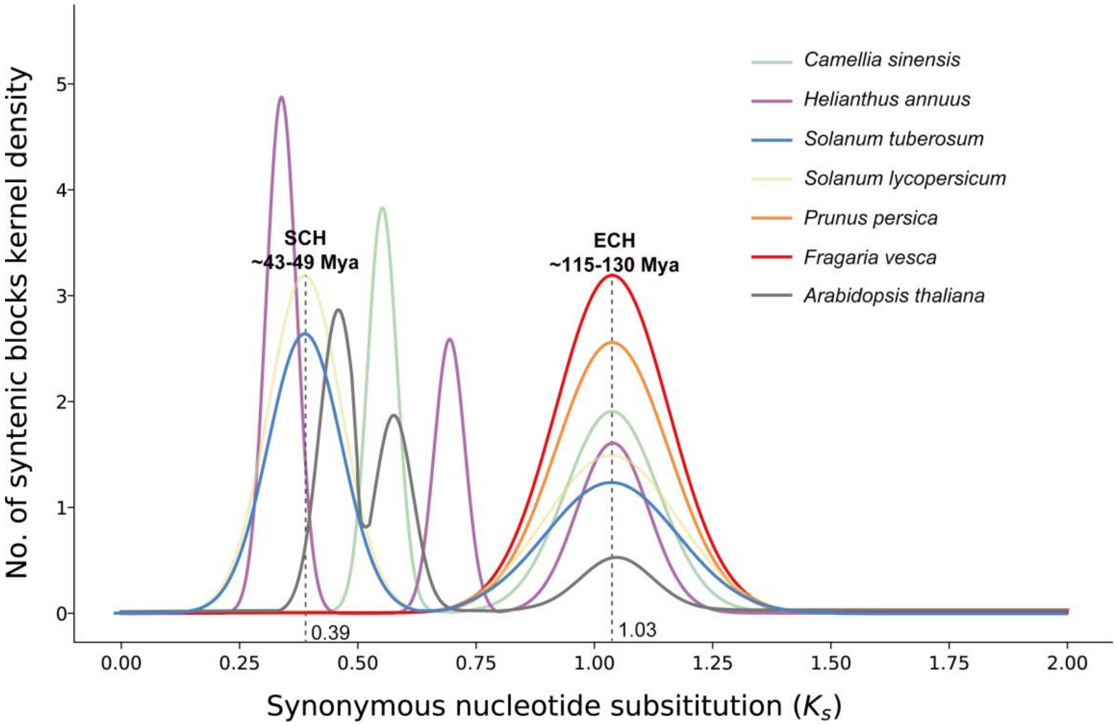

Supplementary Figure 5. Distribution of average synonymous substitutions (*Ks*) between syntenic blocks after evolutionary rate correction. ECH: Eudicot-common hexaploidy; SCH: Solanaceae-common hexaploidization event; Mya: million years ago.

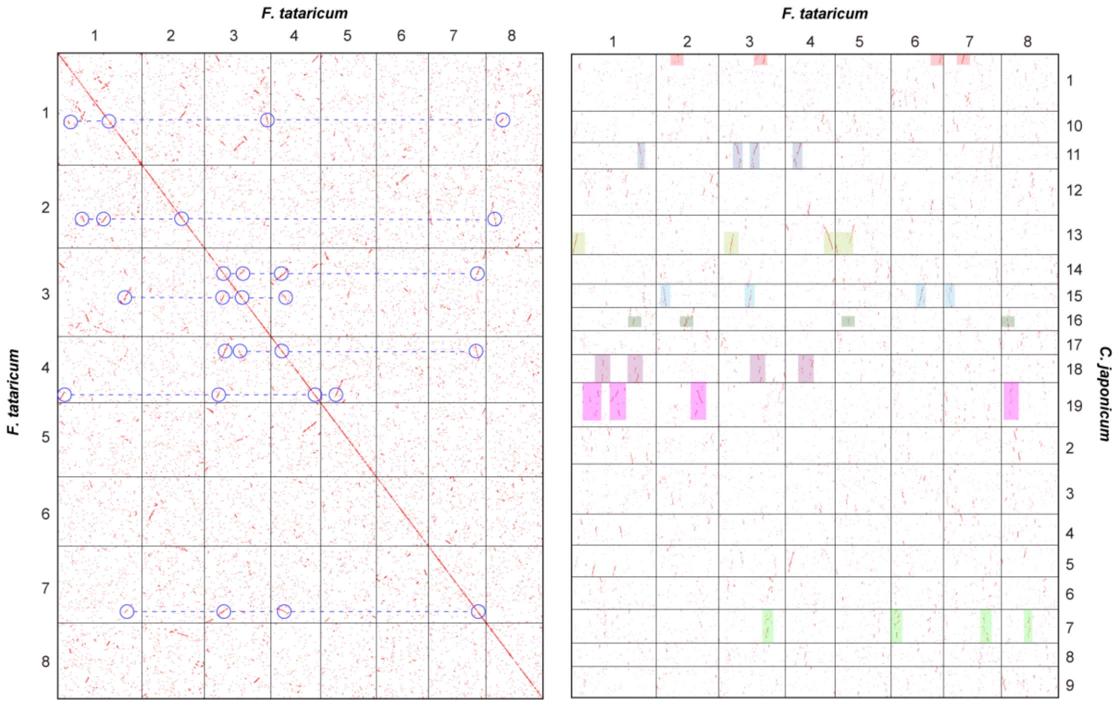

Supplementary Figure 6. Homologous dotplot within *F. tataricum* genome (left) and between selected *C. japonicum* and *F. tataricum* chromosomes (right). The 1:4 chromosomal relationships within *F. tataricum* genome were highlighted in blue circles, and the 1: 4 syntenic block ratio of two species were also highlighted by rectangle (one color corresponding to one chromosome of *C. japonicum*).

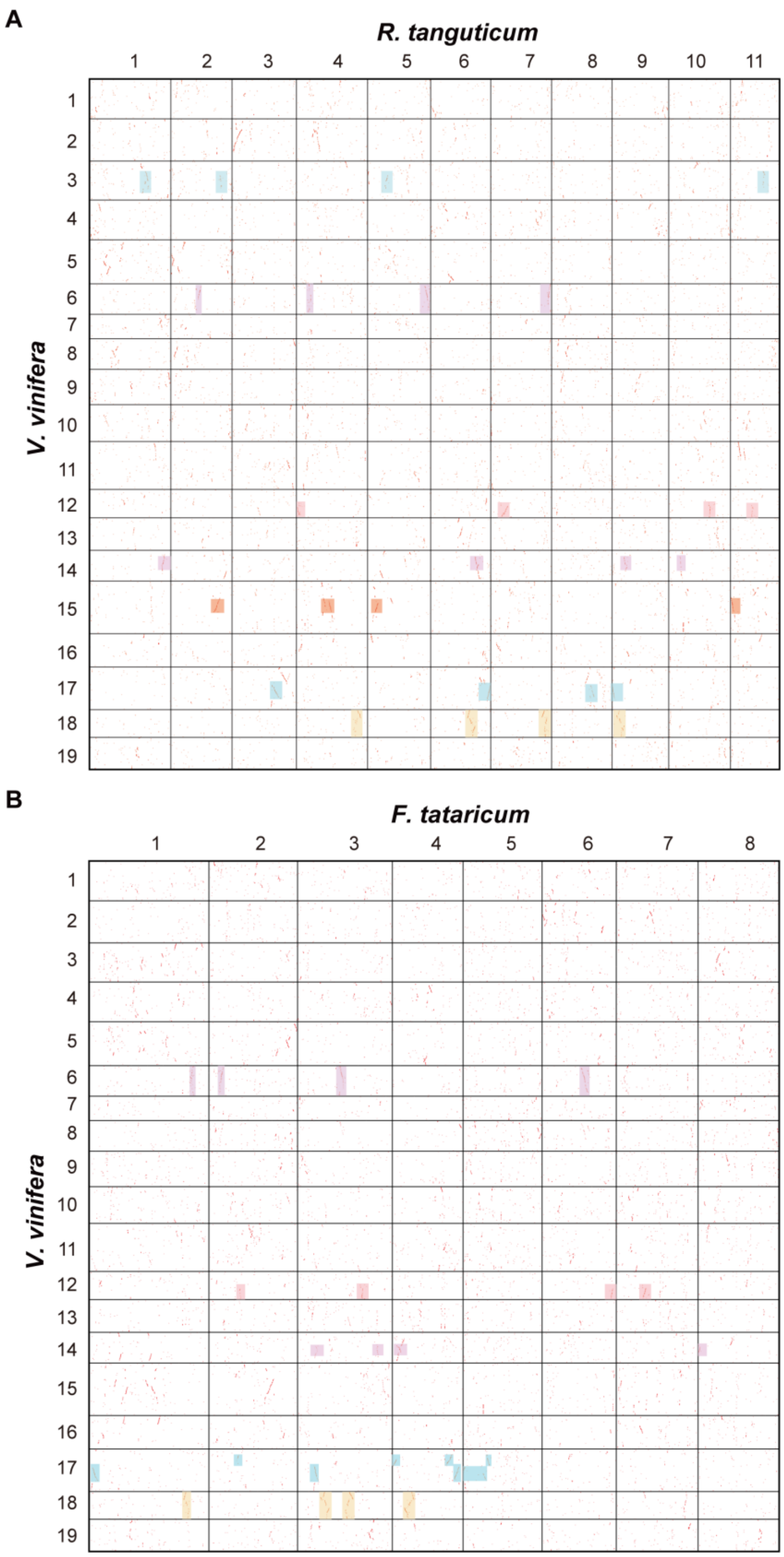

Supplementary Figure 7. Homologous dotplot between selected *V. vinifera* and two Polygonaceae species chromosomes. The highlighted parts (one color corresponding to one chromosome of *V. vinifera*) reveal the 1: 4 syntenic block ratio of two species.

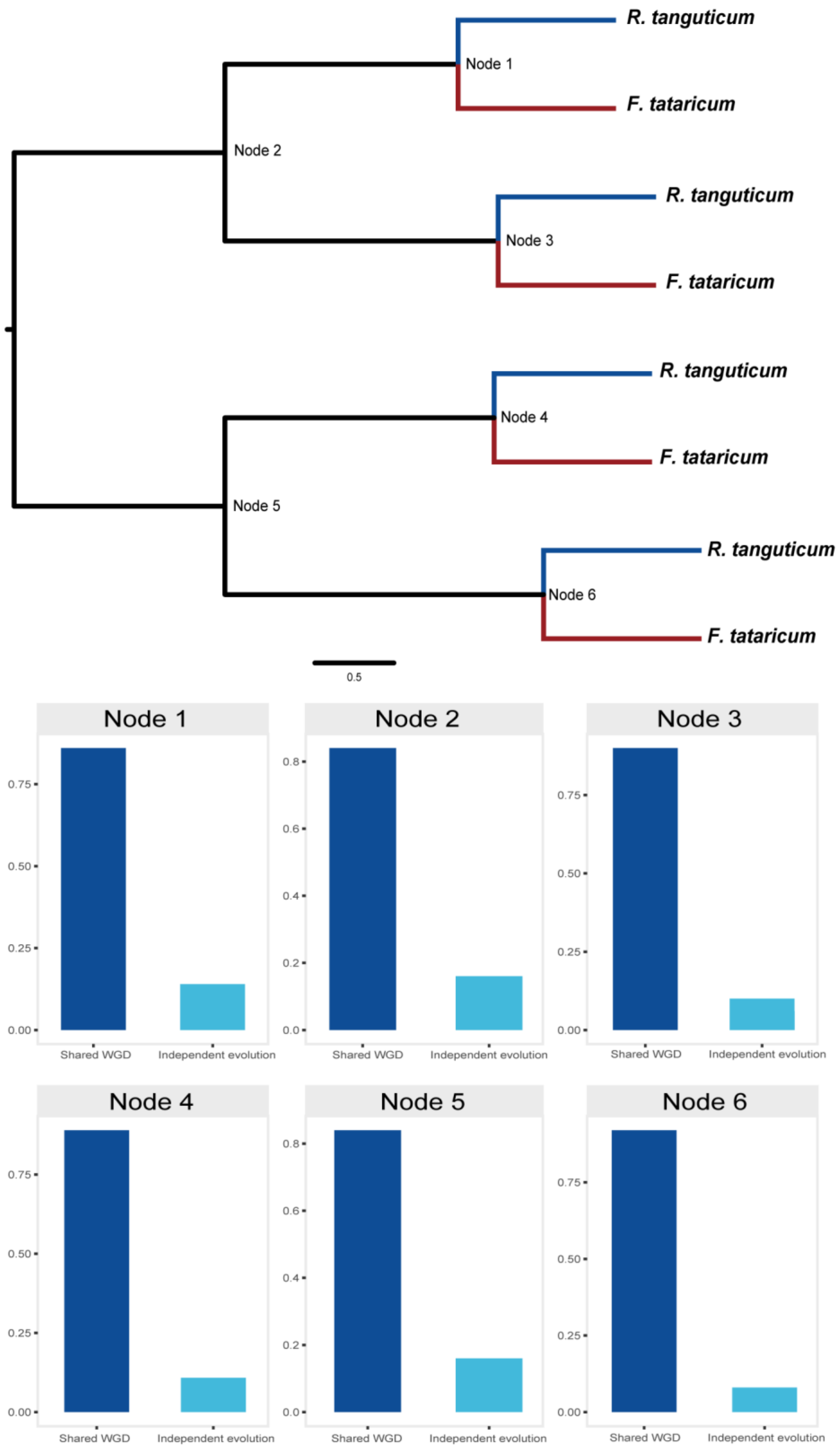

Supplementary Figure 8. The phylogeny between synteny genes of two Polygonaceae species and ASTRAL quartet-score analyses for branches major nodes. Quartet scores were calculated for the two possible evolutionary scenarios (shared WGD and independent evolution) for respective branch. The principal quartet trees (shared WGD) are depicted.

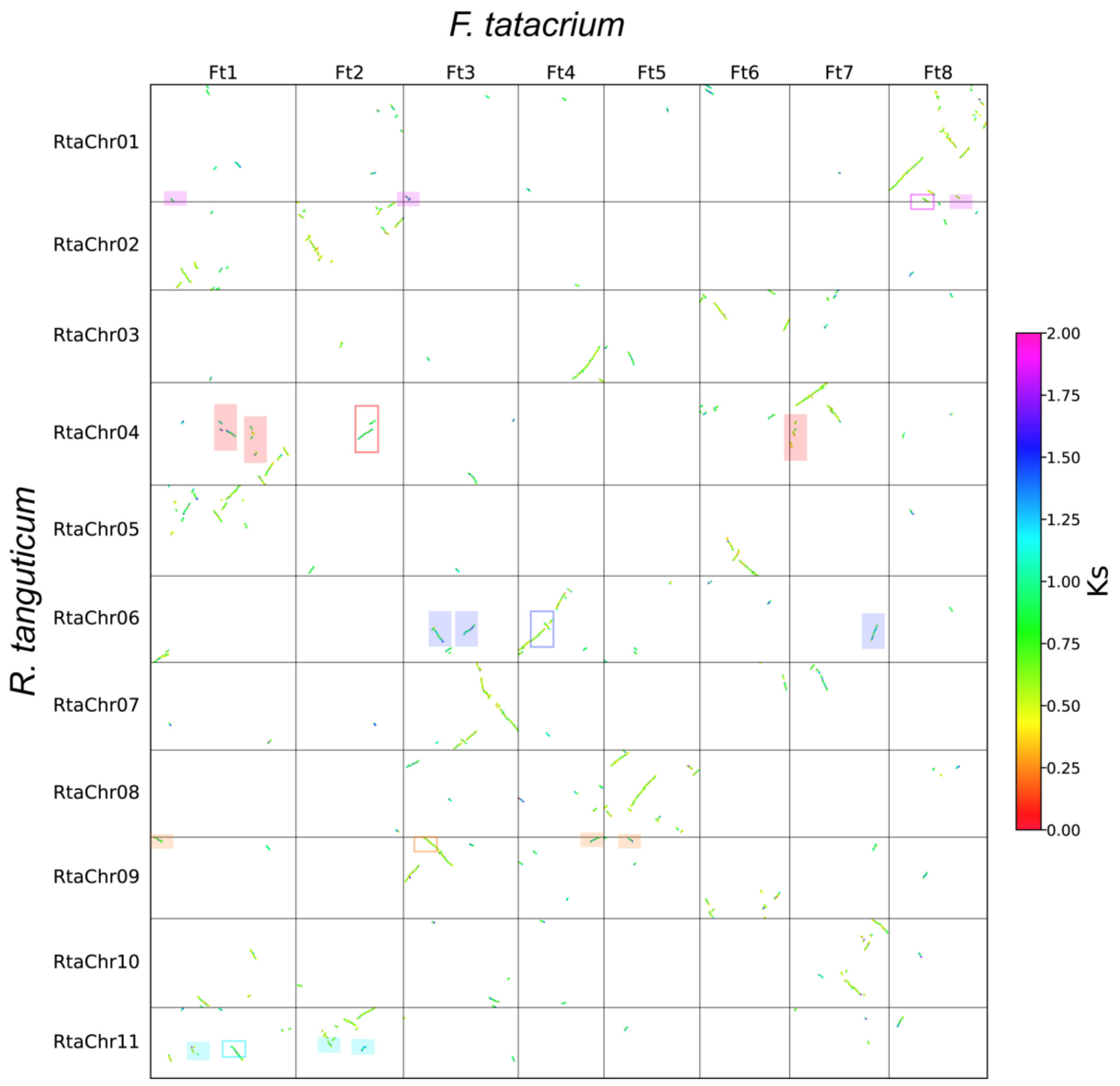

Supplementary Figure 9. Homologous dotplot with *Ks* values between two Polygonaceae species chromosomes. The highlighted parts (one color corresponding to one chromosome of *R. tanguticum*) reveal one closest related (unfilled rectangle) collinear region and three other copied collinear regions in the other species (filled rectangle).

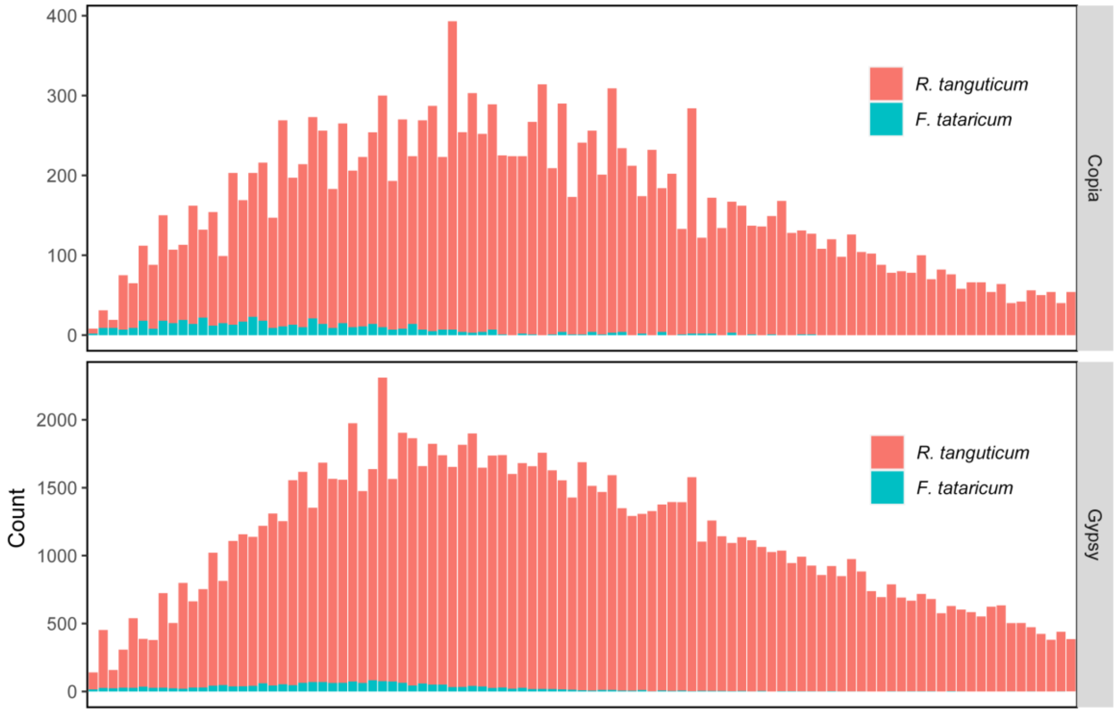

Supplementary Figure 10. Estimation of *Copia* and *Gypsy* insertion time showing a different burst tendency of LTR in *R. tanguticum* and *F. tataricum.* Distribution of insertion times of *Copia* and *Gypsy* elements in the two species.

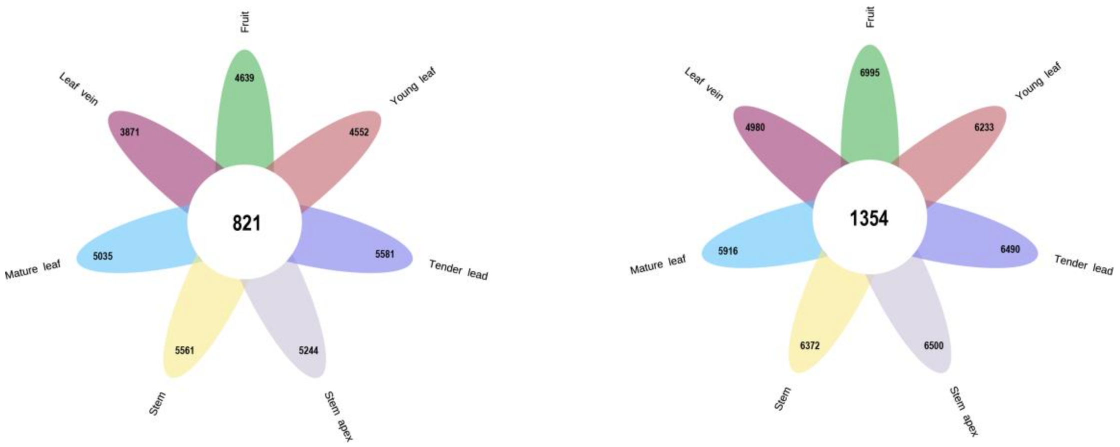

Supplementary Figure 11. Number of different expression genes between root and other tissues. Upregulated (left) and downregulated (right) genes in root compared with other seven tissues, and the number of center is the number of different expression genes that share in all groups.

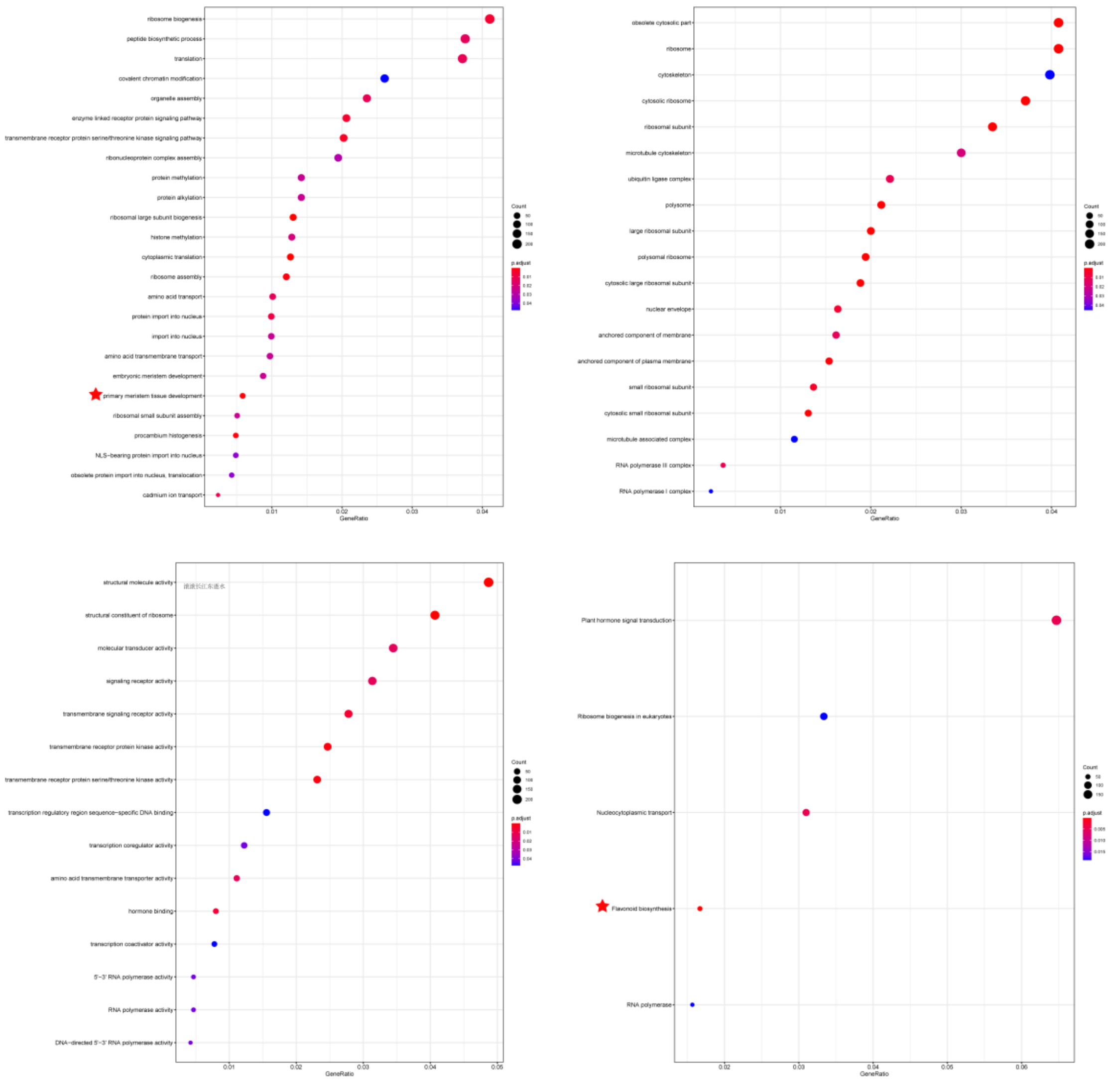

Supplementary Figure 12. Functional enrichment analysis of genes that upregulated in root. The size of the circles represents the number of genes in a GO and KEGG term. The color of the circles represents the statistical significance of enriched GO and KEGG terms. '*P*-adjust' is the adjusted *P*-value of the Benjamini–Hochberg false discovery rate.

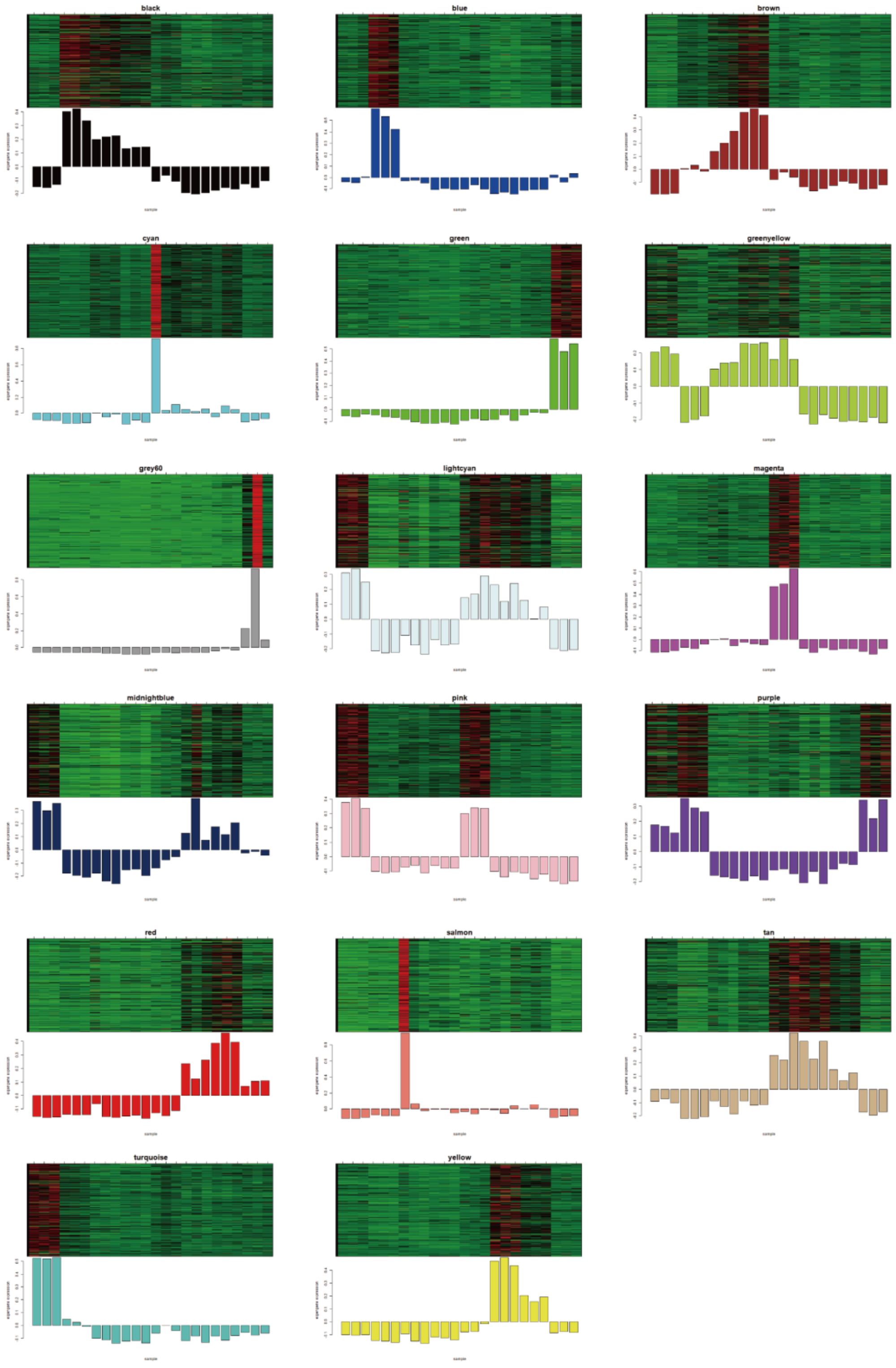

Supplementary Figure 13. Heatmap of gene expression pattern of 18 co-expression modules identified by WGCNA.

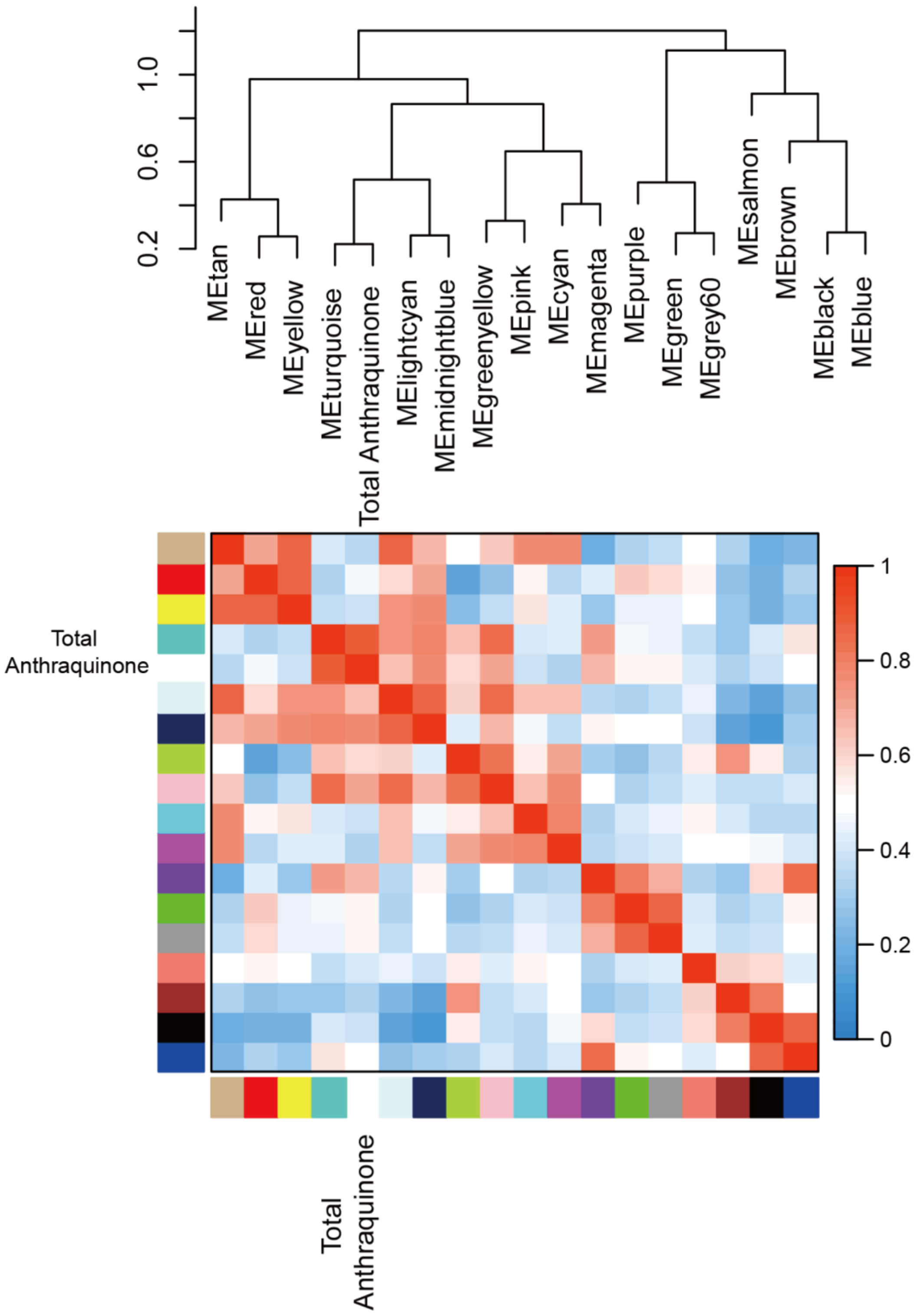

Supplementary Figure 14. Clustering of module eigengenes that summarize the modules found in the clustering analysis. (A) Hierarchical clustering of module eigengenes. Branches of the dendrogram (the meta-modules) group together eigengenes that are positively correlated. **(B)** Heatmap plot of the adjacencies in the eigengene network including the trait weight.

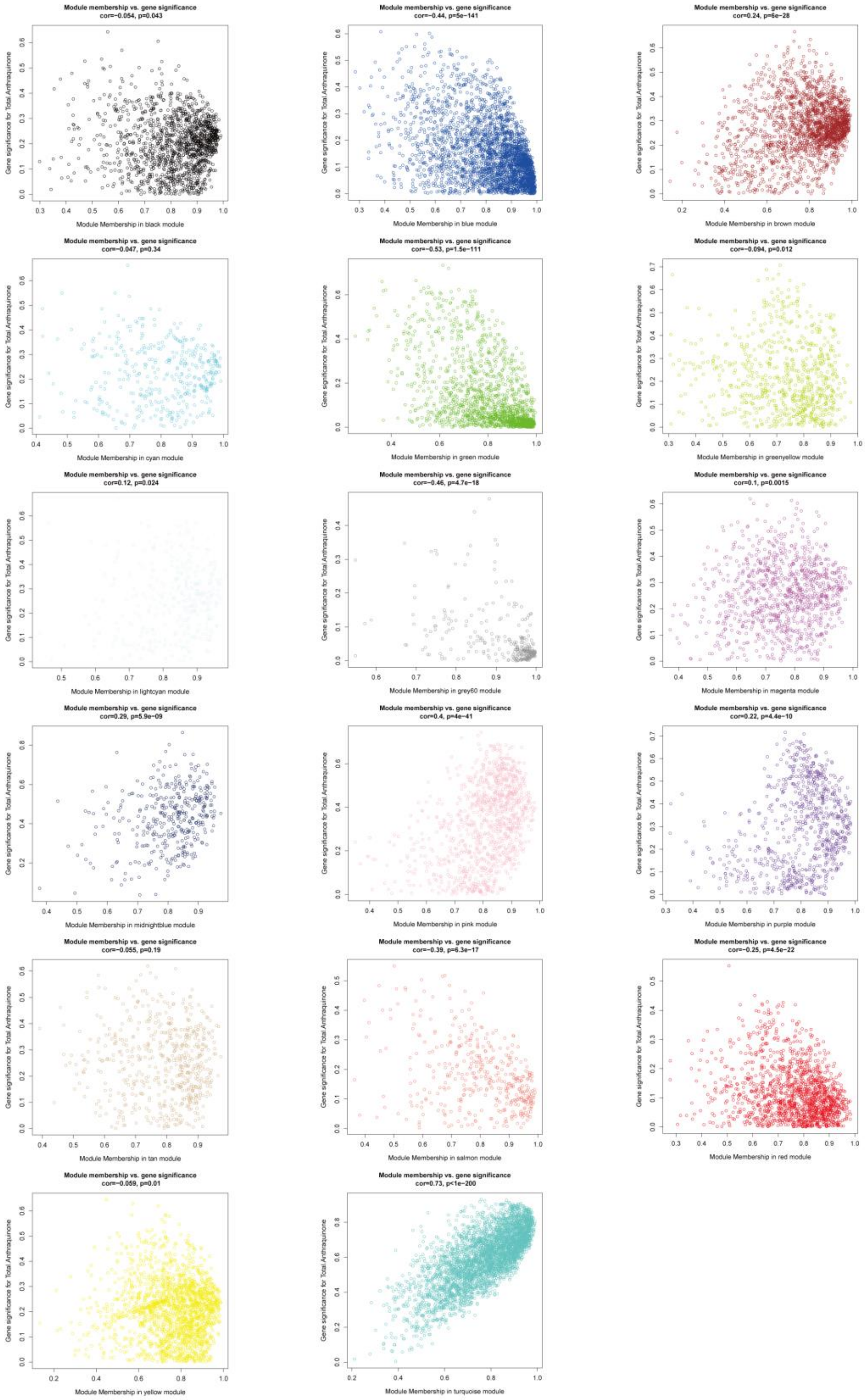

Supplementary Figure 15. Scatter plots of gene significance for weight (GS) versus module membership (MM) in the 18 co-expression modules.

Supplementary Tables

Supplementary Table 1. Summary of the raw sequencing data.

Library Type	Base (Gb)	Depth (×)
Illumina	206.84	75
Nanopore	228.80	84
Hi-C	296.45	108

* Depth (×) = base count / estimated genome size

Supplementary Table 2. Numbers of Illumina short reads mapped to the assembled *R. tanguticum* genome.

Library Type	Total pairs	Pair end mapped reads	Pair end mapped ratio	Single end mapped reads	Single end mapped ratio	Total mapped reads	Total mapped ratio
Illumina	1,335,662,642	1,318,780,156	98.74	807,838	0.06	1,330,856,645	99.64

Supplementary Table 3. Quality assessment of the assembled genome of *R. tanguticum* using QV scores and LAI index.

Chr	QV score	LAI
RtaChr01	27.6045	28.26
RtaChr02	27.9577	27.59
RtaChr03	27.8336	27.87
RtaChr04	27.5455	27.32
RtaChr05	27.6751	27.05
RtaChr06	27.5783	27.04
RtaChr07	27.9215	27.15
RtaChr08	27.7673	26.57
RtaChr09	27.8923	27.65
RtaChr10	28.0081	26.11
RtaChr11	27.6507	28.93

Supplementary Table 4. Quality assessment of the assembled genome of *R. tanguticum* using BUSCOs.

**Type**	**Number**	**Percent (%)**
Complete BUSCOs (C)	248	97.3
Complete and single-copy BUSCOs (S)	178	69.8
Complete and duplicated BUSCOs (D)	70	27.5
Fragmented BUSCOs (F)	4	1.2
Missing BUSCOs (M)	4	1.5
Total BUSCO groups searched	255	100

Supplementary Table 5. Summary of chromosome-level assembles of *R.tanguticum* based on Hi-C data.

Species	Chromosome	Number of anchored and oriented contigs	Length of anchored and oriented contigs (bp)	Number of anchored and oriented gene
R.tanguticum	Chr1	251	303,179,193	5,613
	Chr2	259	259,362,129	4,223
	Chr3	181	252,490,892	4,442
	Chr4	212	247,517,156	4,909
	Chr5	202	245,346,884	4,353
	Chr6	207	245,343,095	4,147
	Chr7	181	242,862,938	4,199
	Chr8	219	240,708,114	4,168
	Chr9	169	240,013,100	3,905
	Chr10	202	239,847,201	4,260
	Chr11	161	194,260,622	3,393

Supplementary Table 6. Prediction of protein-coding genes in the *R. tanguticum* genomes.

Gene set		Total Genes Predicted	Average Gene Length (bp)	Average CDS Length (bp)	Average Exons per Gene	Average Exon Length (bp)	Average Intron Length (bp)
De novo	augustus	63756	2363.83	864.46	4.57	189.04	419.45
	genescan	53413	30918.98	1085.34	5.98	181.37	5985.65
	glimmerhmm	73845	1297.12	612.14	2.87	213.45	366.71
Homolog	*Arabidopsis thaliana*	28569	3076.41	1175.49	5.47	214.81	425.05
	Beta vulgaris	40681	2726.35	1232.06	4.47	275.55	430.48
	Prunus persica	30812	3048.32	1242.65	5.23	237.56	426.79
	Spinacia oleracea	38792	2793.95	1239.02	4.60	269.20	431.62
	Vitis vinifera	30953	3049.74	1211.51	5.23	231.55	434.34
	Fagopyrum tataricum	45230	3115.11	1161.90	5.00	232.45	488.48
RNA-seq		28678	4401.31	1157.68	5.04	229.61	517.81
EVM		49000	5486.31	976.85	5.06	193.12	1111.16
**Final**		**31898**	**3961.62**	**1099.19**	**5.43**	**202.29**	**645.63**

Supplementary Table 7. Quality assessment of the gene annotations of *R. tanguticum* using BUSCOs.

**Type**	***R. tanguticum***	
	**Number**	**Percent (%)**
Complete BUSCOs (C)	237	92.9
Complete and single-copy BUSCOs (S)	176	69.0
Complete and duplicated BUSCOs (D)	61	23.9
Fragmented BUSCOs (F)	10	3.9
Missing BUSCOs (M)	8	3.2
Total BUSCO groups searched	255	100

Supplementary Table 8. Functional annotation of predicted protein-coding genes in the *R. tanguticum* genome.

Database	Number of genes annotated	Percentage (%)
GO	23278	72.98
KEGG	23035	72.21
SWISS-PROT	24107	75.58
TrEMBL	29374	92.09
NR	29412	92.21
Total	31743	95.6

Supplementary Table 9. Detection of transcription factors in the *R. tanguticum* genomes.

**Transcription factor**	**Number**	**Transcription factor**	**Number**	**Transcription factor**	**Number**
LFY	1	RB	1	SET	62
HSF	0	HB-KNOX	10	B3-ARF	34
CAMTA	1	DDT	5	TRAF	37
MADS-MIKC	3	MYB	146	SWI/SNF-SWI3	6
MADS-M-type	11	SNF2	44	Others	101
VOZ	3	Tify	16	mTERF	58
FAR1	25	GNAT	45	TAZ	9
LIM	4	PHD	42	B3	94
SAP	2	NAC	112	BES1	15
C2C2-LSD	3	STAT	1	BBR-BPC	8
DBB	3	EIL	9	OFP	31
TCP	16	NF-YA	12	LUG	7
zf-HD	13	SBP	22	HMG	19
Coactivator	4	SWI/SNF-BAF60b	21	HB-other	20
Pseudo	5	WRKY	86	bZIP	120
DBP	1	bHLH	155	C3H	104
C2C2-CO-like	10	GeBP	15	ULT	2
NF-YB	14	AP2/ERF-ERF	145	SRS	15
HB-BELL	11	AP2/ERF-AP2	21	ARID	24
GRF	9	MED7	1	Alfin-like	12
HB-PHD	2	C2C2-Dof	39	AP2/ERF-RAV	7
Whirly	2	HB-HD-ZIP	48	E2F-DP	15
GARP-ARR-B	10	SOH1	1	MYB-related	132
C2C2-YABBY	8	GRAS	56	TUB	26
HB-WOX	14	PLATZ	17	Rcd1-like	9
CSD	2	Trihelix	40	NOZZLE	0
BSD	1	GARP-G2-like	57	AUX/IAA	74
RWP-RK	13	C2C2-GATA	36	MED6	3
IWS1	11	MBF1	4	S1Fa-like	5
NF-YC	10	NF-X1	5	CPP	23
LOB	46	Jumonji	24	**Total**	**2567**
HRT	1	C2H2	137		

Supplementary Table 10. Prediction of noncoding RNAs in the *R. tanguticum* genomes.

Type	Total number	Average length (bp)	Total length (bp)
antisense	7	190.43	1333
miRNA	130	128.19	16,665
rRNA	4593	167.05	767,283
ribozyme	8	111.50	892
sRNA	4	200.75	803
snRNA	1,262	114.23	144,162
tRNA	4,106	71.84	294,982

Supplementary Table 11. Classification of gene duplicates origin in *R. tanguticum* genome.

Type	Number of genes	Number of genes overlap with Expand gene family	Ratio of genes overlap with Expand gene family
Expand	6,077	6,077	100.0%
WGD	5,806	2,534	41.7%
TD	1,747	705	8.6%
PD	1,274	408	6.7%
TRD	6,587	852	14.0%
DSD	12,756	1,651	27.2%

Supplementary Table 12. Statistics of the number of the gene pair with *Ks* value in different comparation.

Groups	Number of gene pair with *Ks* value
R. tanguticum-R. tanguticum	9,963
F. tataricum-F. tataricum	7,906
S. oleracea -S. oleracea	3,336
B. vulgaris-B. vulgaris	3,084
V. vinifera-V. vinifera	9,031
R. tanguticum-F. tataricum	21,653
R. tanguticum-V. vinifera	10,986
R. tanguticum-C. japonicum	13,153

Supplementary Table 13. Summary statistics of the annotated transposable elements in the *R. nobile and R. tanguticum* genome.

Type	*R. tanguticum*		
	**Length(bp)**	**% of repeat**	**% of genome**
**SINE**	**795666**	**0.03**	**0.03**
**LINE**	**72889523**	**3.03**	**2.64**
L1	70536559	2.93	2.55
L2	722935	0.03	0.03
LINE other	1754931	0.07	0.06
**LTR**	**1980877346**	**82.31**	**71.72**
Copia	272697030	11.33	9.87
Gypsy	1394789010	57.96	50.50
other	408217936	16.96	14.78
**DNA**	**113937200**	**4.74**	**4.13**
CMC-EnSpm	15072925	0.63	0.55
DNA other	99079537	4.12	3.59
Unclassified ARTEFACT	1073	0.00	0.00
Unclassified Other/Composite	204	0.00	0.00
Unclassified RC	3651	0.00	0.00
Unclassified RC/Helitron	16128308	0.67	0.58
Unclassified Retroposon	806	0.00	0.00
Unclassified Retroposon/L1	643	0.00	0.00
Unclassified Unknown	277657971	11.54	10.05
**Satellite**	**3041100**	**0.13**	**0.11**
**Simple repeat**	**146899269**	**6.10**	**5.32**
**Small RNA**	**479979**	**0.02**	**0.02**
**Total**	**2406518263**	**100.00**	**87.13**
**Low complexity**	**1255793**	**0.05**	**0.05**

Supplementary Table 14. Statistics of Illumina short reads of each transcriptome sample mapped to the assembled *R. tanguticum* genome.

Sample ID	Number of bases	Number of reads	Number of mapped reads	Mapped ratio (%)
Root1	7085510902	53949628	50455707	93.52
Root2	7049662372	53938220	50330248	93.31
Root3	7029363514	53317365	49898992	93.59
Tender leaf1	7166722681	53644667	50391599	93.94
Tender leaf2	6952151498	52137039	48989192	93.96
Tender leaf3	7164473404	53496378	50272589	93.97
Young leaf1	6962383298	51719012	48637322	94.04
Young leaf2	6998813801	52306464	49508922	94.65
Young leaf3	7000472704	52056759	49091307	94.3
Mature leaf1	7083664460	52472387	49443404	94.23
Mature leaf2	6984275509	51491672	48532322	94.25
Mature leaf3	6990625830	51867736	48979963	94.43
Leaf vein1	7190229142	54655165	51407936	94.06
Leaf vein2	6962929909	51834346	48675419	93.91
Leaf vein3	7062472847	52176661	48719118	93.37
Stem1	6196087268	44918012	41635270	92.69
Stem2	6003379212	46135300	42886509	92.96
Stem3	6112249002	46210202	42918962	92.88
Stem apex1	6180167941	46430903	42476799	91.48
Stem apex2	6168849445	46088171	41848568	90.8
Stem apex3	6174934380	45881746	41745947	90.99
Fruit1	6018188474	49625220	46396745	93.49
Fruit2	6006812728	55026186	51659980	93.88
Fruit3	6179909397	46794634	43319018	92.57

Supplementary Table 15. Identification of candidate gene families in the ‘turquoise’ module that with the highest correlation with total anthraquinone content.

Gene family	Genes cluster in turquoise module	Hub genes
CHS	RtaG0007463.1,RtaG0027053.1, RtaG0007460.1,RtaG0008681.1, RtaG0007464.1,RtaG0026982.1, RtaG0008012.1	RtaG0007463.1
CYP450	RtaG0020112.1,RtaG0014375.1, RtaG0023526.1,RtaG0010742.1, RtaG0014376.1,RtaG0004593.1, RtaG0030644.1,RtaG0001842.1, RtaG0026174.1,RtaG0020111.1, RtaG0008258.1,RtaG0020560.1, RtaG0003750.1,RtaG0016923.1, RtaG0020614.1,RtaG0029997.1, RtaG0022484.1,RtaG0029845.1, RtaG0000586.1,RtaG0008358.1, RtaG0000350.1,RtaG0007134.1, RtaG0028342.1,RtaG0018310.1, RtaG0009500.1,RtaG0000585.1, RtaG0023880.1,RtaG0000587.1, RtaG0007135.1	RtaG0030644.1, RtaG0014375.1, RtaG0014376.1, RtaG0026174.1
BGL	RtaG0009186.1,RtaG0022724.1, RtaG0017493.1	RtaG0009186.1,RtaG0022724.1

Supplementary Table 16. Reference information of the genomes used in this study.

Species	Version	Source
Beta vulgaris	RefBeet-1.2.2	NCBI
Solanum oleracea	v1.0	NCBI
Fagopyrum tataricum	Pinku1	http://mbkbase.org/Pinku1
Simmondsia chinensis	v1.0	ftp://download.big.ac.cn
Fragaria vesca	v4.0.a2	ftp://ftp.bioinfo.wsu.edu
Prunus persica	v2	NCBI
Arabidopsis thaliana	TAIR10.1	NCBI
Vitis vinifera	12X	NCBI
Camellia sinensis	HZAU_G240_1.0	NCBI
Solanum lycopersicum	v3.0	NCBI
Solanum tuberosum	v3.0	NCBI
Helianthus annuus	HanXRQr2.0	NCBI
Oryza sativa	IRGSP-1.0	NCBI
Zea mays	NAM-5.0	NCBI
Rheum tanguticum	v1.0	This study
